# Joint impact of serum urate, renal function, and genetic susceptibility on coronary heart disease and ischemic stroke risk: a population-based study

**DOI:** 10.3389/fendo.2025.1728019

**Published:** 2025-12-12

**Authors:** Huangda Guo, Siyue Wang, Hexiang Peng, Tianjiao Hou, Yixin Li, Hanyu Zhang, Mengying Wang, Tao Wu, Jie Huang

**Affiliations:** 1 Department of Epidemiology and Biostatistics, School of Public Health, Peking University, Beijing, China; 2 Key Laboratory of Epidemiology of Major Diseases (Peking University), Ministry of Education, Beijing, China; 3 Department of Nutrition and Food Hygiene, School of Public Health, Peking University, Beijing, China; 4 School of Public Health and Emergency Management, Southern University of Science and Technology, Shenzhen, China

**Keywords:** serum urate, renal function, genetic risk, cardiovascular diseases, cohort study

## Abstract

**Background:**

The relationship between serum urate and cardiovascular disease (CVD) is well-established, but its interplay with renal function and genetic susceptibility remains less clear. The study aimed to investigate the individual and joint associations of serum urate, renal function, and genetic risk with incident CVD.

**Methods:**

The study included 383,390 participants from the UK Biobank, initially free of CVDs at baseline. Serum urate levels and kidney damage markers were obtained. We used a new approach to construct an estimated glomerular filtration rate, and incorporate albumin-creatinine ratio to assess renal function. Genetic risk scores for CHD and IS were calculated. Hazard ratios (HR) and 95% confidence intervals (CI) were estimated using Cox models.

**Results:**

Over a median 13.24-year follow-up, 35,932 CVD events were documented, including 30,025 CHD and 5,524 IS cases. Each standard deviation increase in urate was associated with HRs (95% CIs) of 1.09 (1.08, 1.11) for CVD, 1.08 (1.08, 1.11) for CHD, and 1.12 (1.08, 1.15) for IS. Elevated urate, in combination with impaired renal function or higher genetic risk, further increased CVDs risk. Participants with poor renal function and the highest tertile urate had approximately three times the risk of CVDs compared to those with normal kidney function and the lowest urate tertile. Similar trends were observed for the joint impact of genetic susceptibility and urate.

**Conclusions:**

Our findings underscore the importance of managing urate levels in individuals with renal impairment or genetic susceptibility in the prevention of CVDs.

## Graphical Abstract

**Figure:**
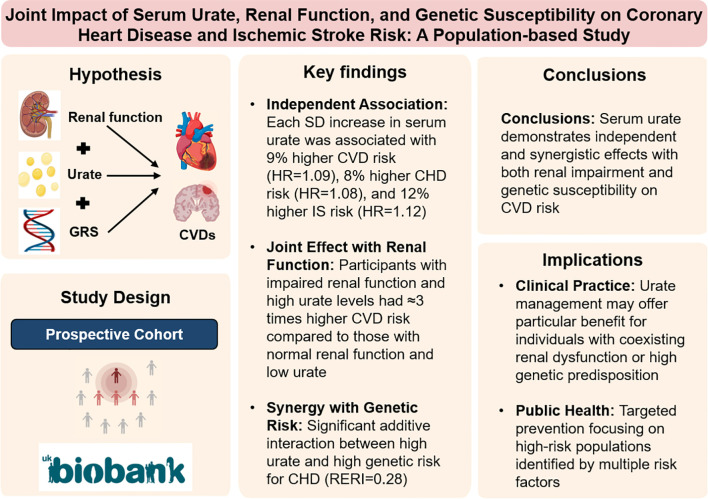
Overview of the study. CHD, coronary heart disease; CI, confidence interval; CVD, cardiovascular disease; GRS, genetic risk score; HR, hazard ratio. IS, ischemic stroke; RERI, relative excess risk due to interaction.

## Highlights

**What is new?**

Our findings indicate that elevated urate, combination of impaired kidney function and raised urate, as well as combination of elevated urate and greater genetic risk significantly increase the risk of cardiovascular diseases.

**What are the clinical implications?**

Our findings demonstrate the importance of lowering urate for the prevention of cardiovascular disease in renally impaired and genetically susceptible populations.

## 1 Introduction

Cardiovascular diseases (CVDs), primarily coronary heart disease (CHD) and ischemic stroke (IS), is the leading cause of death worldwide and the main contributor to disability (1). With changing lifestyles and an aging population, the prevalence of hyperuricemia and gout is increasing worldwide (2). While numerous observational studies have linked elevated serum urate levels with increased CVD risk (3–5), the causal nature of this relationship is intensely debated. Critically, evidence from Mendelian randomization (MR) studies has yielded conflicting conclusions. Some MR analyses suggest no causal effect of urate on coronary artery disease or atrial fibrillation (6), while others report a causal link, particularly for CHD and myocardial infarction in specific subgroups (7). Given these inconsistencies, it is crucial to move beyond simple causal assumptions and explore more complex pathways, such as how urate may interact with established risk factors like renal function, to better understand its role in cardiovascular health.

A number of epidemiological studies have documented the association of decreased renal function with increased risks of CVD and death (8, 9). Reflecting the close ties between renal and cardiovascular health, the American Heart Association (AHA) recently introduced the concept of cardiovascular-kidney-metabolic (CKM) health, further highlighting the interrelation between kidney health and the health of the cardiovascular system (10). Hyperuricemia is prevalent in patients with chronic kidney disease (CKD) (11), and both conditions are frequently observed in individuals with CVD (3–5, 8, 9). Nevertheless, few studies have jointly examined elevated urate levels and impaired renal function in relation to CVD risk. It therefore remains unclear whether the presence of CKD alters the association between urate and cardiovascular outcomes.

Beyond established risk factors such as renal impairment, genetic predisposition also plays a fundamental role in the development of CVDs. It is generally accepted that both genetic and environmental factors contribute to the development of CVDs. A polygenic risk score (GRS) aggregates the effects of many genetic variants across the genome into a single measure, providing a quantitative indicator of an individual’s inherited predisposition to a specific disease (12). In recent years, several studies have suggested that genetic susceptibility (eg. GRS) may interact with lifestyle factors in cardiometabolic outcomes (13, 14). However, it remains unclear whether urate-lowering interventions can counteract the genetically elevated risk of CVDs, and whether the cardiovascular benefit of lower urate levels varies by genetic risk.

Therefore, this study aimed to move beyond the unresolved question of causality and focus on clarifying these complex interrelationships. Using multidimensional prospective cohort data from the UK Biobank, we prospectively assessed the association between elevated serum urate and the risk of CVDs (total CVD, CHD and IS). In particular, we sought to investigate the joint associations of urate levels and renal function, urate and genetic susceptibility, with CVD outcomes. Moreover, we aimed to explore potential interactions of urate with renal function as well as with genetic susceptibility.

## 2 Materials and methods

### 2.1 Study design and population

The study design and methods of UK Biobank have been reported in detail previously (12). In short, the UK Biobank is a large-scale prospective study that recruited over 500,000 participants between the ages of 37–73 from 2006-2010. Participants provided information about lifestyles and other health-related aspects through touch-screen questionnaires and physical measurements. Blood samples were collected for biological testing and genotyping. The UK Biobank study was approved by the Northwest Multicenter Research Ethics Committee (21/NW/0157). Written informed consent was obtained from all participants. The study conformed to the principles outlined in the Declaration of Helsinki.

In the present study, we excluded participants with CHD (n=19,317), stroke (n=8,009), cancer (n=31,202), missing serum urate values, creatinine values, and cystatin C values (n=36,443), and missing covariates (n=29,349) at baseline, leaving 383,390 participants for primary analysis. In analyses exploring the association between albumin-creatinine ratio (ACR) and CVDs, we excluded those without ACR values at baseline, leaving 113,840 participants. In the analyses exploring the interaction between genetic risk score and serum urate, we included only descendants with European ancestry (n=343,054).

### 2.2 Assessment of serum urate and renal function

Serum urate concentrations were obtained by biochemical assays at baseline. The distribution of baseline serum urate was shown in **Supplementary Figure S1**. Specifically, we used estimated glomerular filtration rate (eGFR) separately, or ACR separately, or a combination of both (eGFR & ACR), to evaluate the stage of renal function. Lower eGFR values and higher ACR values indicate more severe renal impairment (13).

The eGFR was calculated using a previously reported equation based on serum creatinine and cystatin C (eGFRcr-cys, unit: mL/min/1.73m^2^), which is considered to be more accurate and led to smaller differences between the Black participants and non-Black participants compared to other methods of estimating GFR (14), specified as follows:


$$\text{eGFRcr}-\text{cys }=\;135\;\times \text{min}{\left(\text{Scr}/\text{k},1\right)}^{{\text{a}}_{1}}\times \text{max}{\left(\text{Scr}/\text{k},1\right)}^{{\text{a}}_{2}}\times \text{min(Scys}/0.8,1{)}^{{\text{b}}_{1}}\times \text{max}{\left(\text{Scys}/0.8,1\right)}^{{\text{b}}_{2}}\times {\text{c}}^{\text{Age}}\times 0.963[\text{if female}]$$

In the equation, Scr refers to the creatinine concentration and Scys refers to the cystatin C concentration. The coefficient ${\text{a}}_{1}$ is used for male participants with creatinine levels less than or equal to 0.9 mg/dL and female participants with creatinine levels less than or equal to 0.7 mg/dL. Coefficient ${\text{a}}_{2}$ is used for male participants with creatinine levels greater than 0.9 mg/dL and female participants with creatinine levels greater than 0.7 mg/dL. The coefficient ${\text{b}}_{1}$ is for cystatin C levels less than or equal to 0.8 mg/L and the coefficient ${\text{b}}_{2}$ is for cystatin C levels greater than 0.8 mg/L. In analyzing the association of eGFRcr-cys with CVD outcomes, we categorized them into five groups based on the KDIGO guideline (13) to reflect different CKD risks: ≥90 (G1 stage), 60-89 (G2 stage), 45-59 (G3a stage), 30-44 (G3b stage), 15-29 (G4 stage), and <15 (G5 stage). When furtherly exploring the joint associations of eGFRcr-cys and urate to each CVD outcome, we divided the eGFRcr-cys into three groups given the sample size limitations of the population at G4 and G5 stages: ≥90 (normal or high), 60-89 (mildly decreased), <60 (moderately to severely decreased) (15).

Albuminuria is another biomarker used for CKD staging. In accordance with the KDIGO guideline (13), we also used ACR (unit: mg/mmol) to reflect the degree of albuminuria in urine and categorized it into three groups, A1-A3: <3 (A1 stage), 3-30 (A2 stage), and >30 (A3 stage).

Furthermore, we defined the risk of renal impairment from a two-dimensional perspective based on the eGFR and ACR categories (13), classifying the risk of the CKD into four groups: low, moderate, high, and very high. The specific categorization was shown in **Supplementary Material** online, **Supplementary Figure S1**.

### 2.3 Construction of genetic risk scores

The genotyping process and arrays used in the UK Biobank study have been described elsewhere (16). For the present study, we constructed separate genetic risk scores (GRS) for CHD and IS. The selection of single nucleotide polymorphisms (SNPs) was based on the recent and large-scale genome-wide association studies (GWAS) of European ancestry for CHD (17) and IS (18). We selected 64 and 32 independent SNPs that reached genome-wide significance (*P* < 5 × 10^-8^). Details of the selected SNPs are provided in **Supplementary Table S1** of the **Supplementary Materials**.

Based on the selected SNPs, the primary weighted GRS was calculated as the sum of risk alleles weighted by their reported effect sizes (*β*-coefficients) from the corresponding GWAS: *GRS_weighted_ = β_1_×SNP_1_+β_2_×SNP_2_+… + β_n_×SNP_n_*, where each SNP was coded as 0, 1, or 2 based on the number of effect alleles. For sensitivity analysis, an alternative standardized weighted GRS was calculated using the formula *GRS_std_* = (Σ (*β_i_* × *SNP_i_*)) ×(*Number of SNPs*)/(Σ *β_i_*) to facilitate interpretation and comparison (**Supplementary Methods**). Participants were then categorized into low (quintile 1), intermediate (quintiles 2-4), or high (quintile 5) genetic risk groups based on the distribution of the primary weighted GRS for each outcome (**Supplementary Figure S1**).

### 2.4 Follow-up and assessment of outcomes

Outcomes were determined from hospital admission records containing admission and diagnosis data obtained from hospital event statistics in England, morbidity record data in Scotland, and patient event databases in Wales. The dates of death for participants were obtained from death certificates provided by the National Health Service Information Center (NHS) for England and Wales and the NHS Central Registry (NHS) for Scotland. Follow-up was terminated by the earliest occurrence of death, loss to follow-up, or the last date for which health data were available.

Diagnoses were recorded using the International Classification of Diseases, 10th Revision (ICD-10) coding system. The primary outcome of the study was the incident CVD and its two primary endpoints including CHD and IS, which were evaluated separately according to ICD-10 defined outcomes: CHD (I20-25), ischemic stroke (I63 and I69.3) and CVD (I20-25, I60-I64, I69).

### 2.5 Covariates

Covariates were documented including age, sex, ethnicity (White, Asian or Asian British, Black or Black British, and Other ethnic groups), qualifications (college or university degree, Advanced [A] levels/Advanced Subsidiary [AS] levels or equivalent, Ordinary [O] levels/General Certificate of Secondary Education [GCSE] or equivalent, Certificate of Secondary Education [CSE] or equivalent, National Vocational Qualification [NVQ] or Higher National Diploma [HND] or Higher National Certificate [HNC] or equivalent, other professional qualifications, or none of the above), income, BMI (kg/m^2^)), smoking status (never, former and current), drinking status (never, former and current), physical activity (MET-h/week), sleep duration (h/day), intake of processed meat, fresh fruits and vegetables, fish, tea and coffee consumption, family history of heart disease or stroke (only in the corresponding analysis), prevalent hypertension, prevalent diabetes, high-density lipoprotein cholesterol (HDLc), low-density lipoprotein cholesterol (LDLc), use of anti-hypertensive drugs, use of anti-hyperlipidemic drugs and use of anti-diabetic drugs. The medication history was based on the response to the touchscreen question “Do you regularly take any of the following medications?” (Field 6153 and 6177). Diabetes was defined according to glycosylated hemoglobin ≥ 6.5%, fasting blood glucose ≥ 7.0 mmol/L, self-reported or use of anti-diabetic drugs. Hypertension was defined as systolic blood pressure (SBP) ≥ 140 mmHg or diastolic blood pressure (DBP) ≥ 90 mmHg, self-reported or on anti-hypertensive medications.

### 2.6 Statistical analysis

Baseline characteristics of 383,390 participants were described as the mean or percentage of urate in each quintile. Hazard ratio (HR) and 95% confidence interval (CI) were estimated using the Cox proportional hazards model. The proportional hazards assumptions of the Cox model were tested using the Schoenfeld residual method and were satisfied. In the primary association analyses, serum urate was modeled both as a continuous variable (per standard deviation increase) and as a categorical variable using quintiles. To assess the potential mediating role of renal function in the association between serum urate and CVD outcomes, we conducted mediation analyses. The analyses estimated the direct effect (DE) of urate, the indirect effect (IE) mediated through eGFRcr-cys and ACR, and the total effect (TE). The proportion mediated was calculated as IE/TE. CIs for the mediation effects were derived using a resampling approach based on 1 million random draws from a multivariate normal distribution (19). For the interaction and joint association analyses, urate was categorized into tertiles to ensure sufficient statistical power. Multiplicative interactions between serum urate (in tertiles) and renal function markers or GRS were tested by introducing cross-product terms into the models and evaluating them using likelihood ratio tests. Additive interactions were assessed by calculating the relative excess risk due to interaction (RERI) and the attributable proportion due to interaction (AP). We further performed joint association analyses to evaluate the combined effects of serum urate (in tertiles) with renal function markers or genetic susceptibility on CVD outcomes. All models were adjusted for the covariates described previously.

### 2.7 Sensitivity analysis

To assess the robustness of our primary findings, we conducted a series of comprehensive sensitivity analyses. For the main association analysis, the following analyses were performed in addition to the primary model: (i) Treating death as a competing risk event using Fine-Gray sub-distribution hazards regression models; (ii) Excluding participants with diabetes at baseline; (iii) Further adjusting for potential environmental stressors, including residential noise pollution, residential particulate air pollution (PM_2.5_ and PM_10_), and self-reported well-being scores; (iv) Excluding participants who developed incident cardiovascular diseases (CVD) within the first two years of follow-up to mitigate potential reverse causation; (v) Performing stratified analyses by self-reported ethnic background (White vs. other ethnic groups) to examine the consistency of associations across different populations; (vi) Additionally adjusting for the use of diuretic medications, as they are common urate-lowering agents and a potential confounder. Furthermore, specifically for the analysis investigating the interaction between the GRS and serum urate, as well as their joint associations with CVDs, we conducted an additional sensitivity analysis using an alternative method (**Supplementary Methods**) for GRS to ensure the reliability of the genetic effect estimates.

Analyses were performed using R (version 4.2.1). A two-sided *P*-value < 0.05 was considered statistically significant. To address potential inflation of type I error due to multiple comparisons, we distinguished between hypothesis-driven and exploratory analyses. The primary associations of urate and renal function markers with cardiovascular outcomes were considered hypothesis-driven and interpreted at the nominal significance level (*P* < 0.05). For exploratory analyses, including the interactions between urate and renal function markers or GRSs, as well as their joint associations with outcomes, we applied Bonferroni correction based on the number of independent tests. The interaction analyses and the joint association analyses involved 11 tests, with a corrected threshold of *P* < 0.0045. Results not surviving correction but with nominal significance (*P* < 0.05) are described as suggestive.

## 3 Results

### 3.1 Baseline characteristics

**Table 1** shows the baseline characteristics of the study participants according to serum urate quintiles. Of the 383,390 participants, those with higher urate levels were older, more likely to be male, smokers, and current drinkers, had higher BMI measurements, higher prevalence of hypertension and diabetes, higher LDL levels and lower HDL levels, as well as poorer renal function assessed by eGFRcr-cys.

**Table 1:** Baseline characteristics by quintiles of serum urate in the UK Biobank cohort.

Characteristic		Serum urate				
		Quintile 1	Quintile 2	Quintile 3	Quintile 4	Quintile 5
No, (%)		76700 (20.00)	76658 (19.99)	76756 (20.02)	76624 (19.99)	76652 (19.99)
Age, mean (SD), y		54.29 (8.18)	55.81 (8.06)	56.51 (7.98)	56.69 (8.02)	56.78 (8.06)
Female (%)		70308 (91.67)	57953 (75.60)	41401 (53.94)	25510 (33.29)	13407 (17.49)
BMI, mean (SD), kg/m^2^		24.90 (3.85)	26.31 (4.25)	27.39 (4.46)	28.32 (4.54)	29.63 (4.72)
Ethnicity (%)	White	69181 (90.20)	69427 (90.57)	69405 (90.42)	69476 (90.67)	69411 (90.55)
	Asian or Asian British	3548 (4.63)	3195 (4.17)	3004 (3.91)	2733 (3.57)	2524 (3.29)
	Black or Black British	460 (0.60)	421 (0.55)	490 (0.64)	447 (0.58)	501 (0.65)
	Other ethnic groups	3511 (4.58)	3615 (4.72)	3857 (5.03)	3968 (5.18)	4216 (5.50)
Qualification (%)	College or University degree	10063 (13.12)	11651 (15.20)	12477 (16.26)	12762 (16.66)	13562 (17.69)
	A levels/AS levels or equivalent	26894 (35.06)	25835 (33.70)	25377 (33.06)	25043 (32.68)	23766 (31.01)
	O levels/GCSEs or equivalent	10488 (13.67)	9588 (12.51)	9324 (12.15)	8993 (11.74)	8996 (11.74)
	CSEs or equivalent	17608 (22.96)	17261 (22.52)	16404 (21.37)	15816 (20.64)	15411 (20.11)
	NVQ or HND or HNC or equivalent	4524 (5.90)	4307 (5.62)	4290 (5.59)	4327 (5.65)	4520 (5.90)
	Other professional qualifications	3286 (4.28)	4028 (5.25)	4926 (6.42)	5881 (7.68)	6650 (8.68)
	None of the above	3837 (5.00)	3988 (5.20)	3958 (5.16)	3802 (4.96)	3747 (4.89)
Current smoker (%)	current	7821 (10.20)	7852 (10.24)	8074 (10.52)	8126 (10.61)	7800 (10.18)
Current drinker (%)	current	69435 (90.53)	70250 (91.64)	70878 (92.34)	71267 (93.01)	72171 (94.15)
Hypertension (%)		28000 (36.51)	33885 (44.20)	38924 (50.71)	43040 (56.17)	47692 (62.22)
Diabetes (%)		3350 (4.37)	4042 (5.27)	4794 (6.25)	5495 (7.17)	6617 (8.63)
HDL cholesterol, mean (SD), mmol/L		1.63 (0.36)	1.56 (0.36)	1.47 (0.35)	1.38 (0.33)	1.30 (0.31)
LDL cholesterol, mean (SD), mmol/L		3.51 (0.81)	3.62 (0.82)	3.66 (0.83)	3.68 (0.83)	3.69 (0.85)
eGFRcr-cys (%)	≥90 mL/min/1.73m^2^	65628 (85.56)	56954 (74.30)	51252 (66.77)	46574 (60.78)	38667 (50.44)
	60–89 mL/min/1.73m^2^	10956 (14.28)	19447 (25.37)	24955 (32.51)	29042 (37.90)	34887 (45.51)
	<60 mL/min/1.73m^2^	116 (0.15)	257 (0.34)	549 (0.72)	1008 (1.32)	3098 (4.04)

A, Advanced; AS, Advanced Subsidiary; BMI, body mass index (calculated as weight in kilograms divided by height in meters squared); CSE, Certificate of Secondary Education; eGFRcr-cys, creatinine and cystatin C-based estimated glomerular filtration rate; GCSE, General Certificate of Secondary Education; HDL, high-density lipoprotein; HNC, Higher National Certificate; HND, Higher National Diploma; LDL, low-density lipoprotein; NVQ, National Vocational Qualification; O, Ordinary; SD, standard deviation.

### 3.2 Serum urate and CVD outcomes

**Figure 1:**
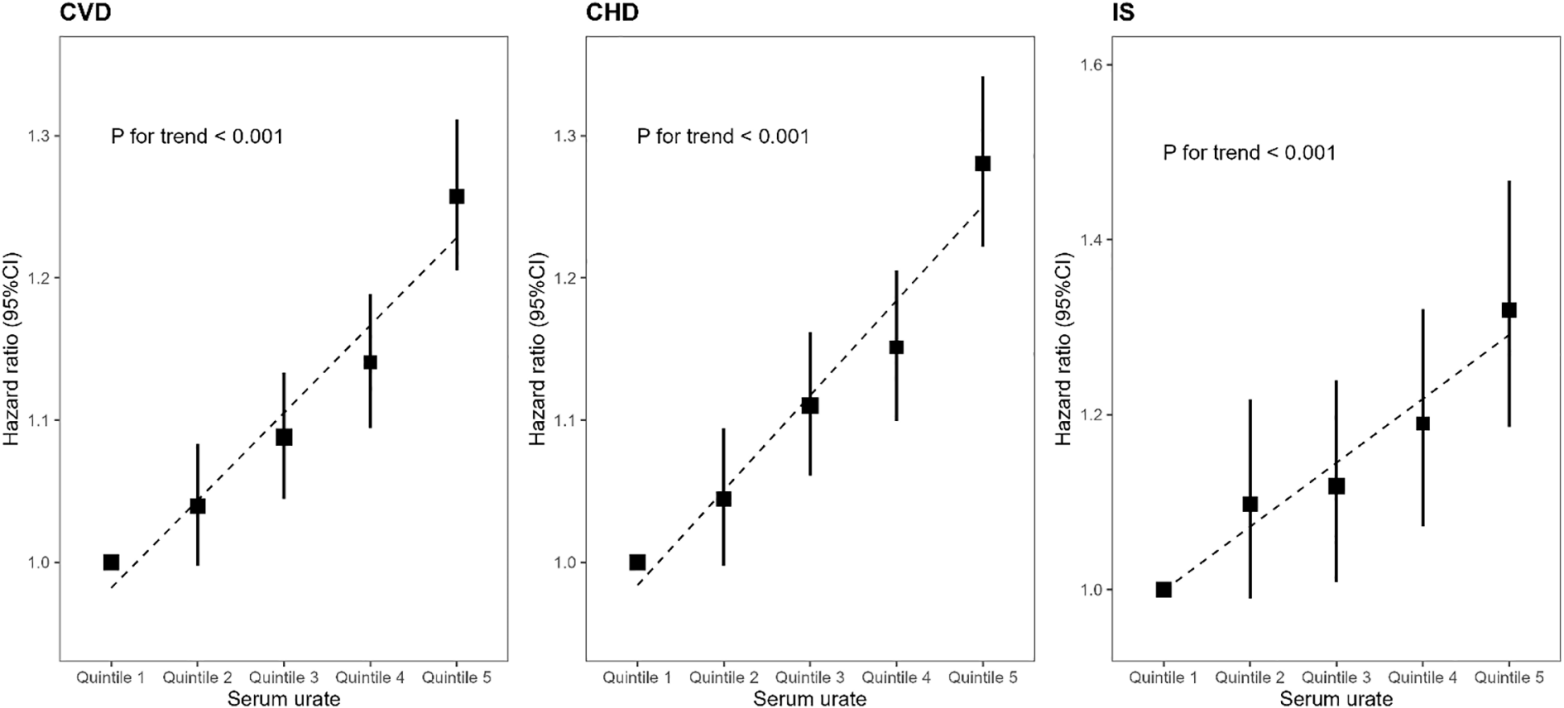
Incident risk of cardiovascular diseases according to serum urate. Multivariable model was adjusted for age, sex, income, ethnic, body mass index(BMI), qualification, smoking status, alcohol status, total physical activity level, duration of sleep, fruit consumption, processed meats consumption, vegetables consumption, fishes consumption, tea consumption, coffee consumption, family history of heart diseases or stroke (only in the corresponding analysis), prevalent hypertension, prevalent diabetes, high-density lipoprotein cholesterol (HDLc), low-density lipoprotein cholesterol (LDLc), use of antihypertensive drugs, use of antihyperlipidemic drugs and use of antidiabetic drugs. CHD, coronary heart disease; CI, confidence interval; CVD, cardiovascular disease; HR, hazard ratio. IS, ischemic stroke.

During a median follow-up period of 13.24 years, we recorded 35,932 cases of CVD, including 30,025 cases of CHD and 5,524 cases of IS. We observed that each standard deviation increase in urate was associated with a 9% (95% CI:8%-11%) increased risk of CVD, 9% (95% CI:8%-11%) higher risk of CHD and 12% (95% CI:8%-15%) higher risk of IS. We observed a significant increase in the risk of CVD, CHD and IS with elevated serum urate (*P* for linear trend <0.001, **Figure 1** and **Supplementary Table S2**). Relative to those in the first quintile of urate, the HRs (95% CIs) for the highest quintile of urate were 1.26 (1.21,1.31) for CVD, 1.28 (1.22,1.34) for CHD, and 1.32 (1.19,1.47) for IS, respectively.

The robustness of these associations was confirmed through a comprehensive set of sensitivity analyses. The results remained virtually unchanged when: (i) treating death as a competing risk using Fine-Gray models (**Supplementary Table S3**); (ii) excluding baseline diabetic patients (**Supplementary Table S4**); (iii) additionally adjusting for environmental stressors and well-being (**Supplementary Table S5**); (iv) excluding participants with incident CVD within the first 2 years of follow-up to minimize reverse causation (**Supplementary Table S6**); (v) stratifying by ethnicity, where the association remained strong and significant in the White population, which constituted the majority of the cohort (**Supplementary Table S7**); and (vi) additionally adjusting for diuretic use, a key urate-lowering medication (**Supplementary Table S8**). In addition, we performed subgroup analyses by age, sex, ethnicity, and smoking status. For each CVD outcome, the association of per 1 standard deviation increase in serum urate appeared to change between subgroups classified by sex, age, or smoking status (**Supplementary Table S9**). For women, aged 60 years or older, and current smokers, the risk of CVD was estimated to be higher.

### 3.3 Kidney damage markers and CVD outcomes

In the present study, we estimated the association of CKD stages defined by two kidney damage markers (eGFRcr-cys and ACR) individually and by them collectively with CVD outcomes. The results were presented in **Table 2**. With a decrease in eGFRcr-cys values, or an increment in ACR values, the risk of CVD, CHD, and IS rose significantly. Compared with participants with eGFRcr-cys ≥ 90 mL/min/1.73m^2^ (G1 stage), the HRs (95% CIs) for CVD, CHD, and IS for participants in G5 stage (eGFRcr-cys < 15) were correspondingly 3.62 (2.56, 5.13), 4.04 (2.85, 5.72), and 3.22 (1.34 7.75). Individuals with ACR >30 mg/mmol (A3 stage) had a 79% (95% CI: 62%, 98%), 75% (95% CI: 56%, 95%), and 117% (95% CI: 74%, 171%) elevated risk of CVD, CHD, and IS, respectively, in comparison to individuals in A1 stage (ACR < 3). When we used the KDIGO guidelines to assess CKD risk by both of the above markers in combination, analogous results of associations with CVDs were also observed. As the CKD risk rose, the risk of all CVD outcomes went up as well. In contrast to those at low risk, populations at high risk for CKD had a greater risk of incident CVD, CHD, and IS at 2.14 (1.90, 2.41), 2.12 (1.87, 2.41), and 2.43 (1.87, 3.17), respectively. These observed associations between renal function markers and CVD outcomes remained consistent across a series of sensitivity analyses (**Supplementary Tables S10-S14**).

**Table 2:** Association of CKD stages (defined by eGFRcr-cys, ACR, or both) with cardiovascular diseases.

CKD stage	CVD		CHD		IS	
	HR (95%CI)	*P*	HR (95%CI)	*P*	HR (95%CI)	*P*
eGFRcr-cys, mL/min/1.73 m^2^						
G1 (≥90)	1.00 (reference)	reference	1.00 (reference)	reference	1.00 (reference)	reference
G2 (60-89)	1.12 (1.10, 1.15)	<0.001	1.11 (1.09, 1.14)	<0.001	1.25 (1.18, 1.33)	<0.001
G3a (45-59)	1.54 (1.44, 1.65)	<0.001	1.50 (1.40, 1.61)	<0.001	1.87 (1.61, 2.18)	<0.001
G3b (30-44)	2.08 (1.85, 2.34)	<0.001	2.07 (1.83, 2.35)	<0.001	2.21 (1.66, 2.94)	<0.001
G4 (15-29)	2.64 (2.15, 3.25)	<0.001	2.38 (1.90, 2.99)	<0.001	3.68 (2.36, 5.72)	<0.001
G5 (<15)	3.62 (2.56, 5.13)	<0.001	4.04 (2.85, 5.72)	<0.001	3.22 (1.34, 7.75)	0.009
ACR, mg/mmol						
A1 (<3)	1.00 (reference)	reference	1.00 (reference)	reference	1.00 (reference)	reference
A2 (3-30)	1.20 (1.15, 1.25)	<0.001	1.18 (1.12, 1.23)	<0.001	1.38 (1.25, 1.53)	<0.001
A3 (>30)	1.79 (1.62, 1.98)	<0.001	1.75 (1.56, 1.95)	<0.001	2.17 (1.74, 2.71)	<0.001
eGFRcr-cys & ACR						
Low	1.00 (reference)	reference	1.00 (reference)	reference	1.00 (reference)	reference
Moderate	1.20 (1.15, 1.25)	<0.001	1.18 (1.13, 1.24)	<0.001	1.29 (1.17, 1.43)	<0.001
High	1.60 (1.46, 1.75)	<0.001	1.52 (1.38, 1.68)	<0.001	2.08 (1.71, 2.53)	<0.001
Very high	2.14 (1.90, 2.41)	<0.001	2.12 (1.87, 2.41)	<0.001	2.43 (1.87, 3.17)	<0.001

Multivariable model was adjusted for age, sex, income, ethnic, body mass index(BMI), qualification, smoking status, alcohol status, total physical activity level, duration of sleep, fruit consumption, processed meats consumption, vegetables consumption, fishes consumption, tea consumption, coffee consumption, family history of heart diseases or stroke (only in the corresponding analysis), prevalent hypertension, prevalent diabetes, high-density lipoprotein cholesterol (HDLc), low-density lipoprotein cholesterol (LDLc), use of antihypertensive drugs, use of antihyperlipidemic drugs and use of antidiabetic drugs. ACR, albumin-to-creatinine ratio; CHD, coronary heart disease; CI, confidence interval; CVD, cardiovascular disease; eGFRcr-cys, creatinine and cystatin C-based estimated glomerular filtration rate; HR, hazard ratio. IS, ischemic stroke.

### 3.4 Mediating effects of renal function on the urate-CVD associations

We conducted mediation analyses to assess the extent to which the association between serum urate and incident CVDs was mediated by renal function, as quantified by eGFRcr-cys and ACR. The results are summarized in **Supplementary Table S15**. A substantial proportion of the total effect of urate on CVD risk was mediated by eGFRcr-cys. The proportion mediated was 43.6% for CVD, 42.6% for CHD, and 51.8% for IS, with all indirect effects being statistically significant. In contrast, the mediation effect through ACR was markedly smaller. The proportion of the total effect mediated by ACR was approximately 2.5% for CVD, 2.3% for CHD, and 2.1% for IS.

### 3.5 Joint association of urate and renal function with CVD outcomes

Given the established role of renal function as a significant mediator, we next investigated the joint effects of serum urate and renal function on CVD risk. We assessed the combined associations of urate with eGFRcr-cys, urate with ACR, and urate with composite CKD risk (defined by both eGFRcr-cys and ACR) in relation to CVD outcomes.

**Figure 2:**
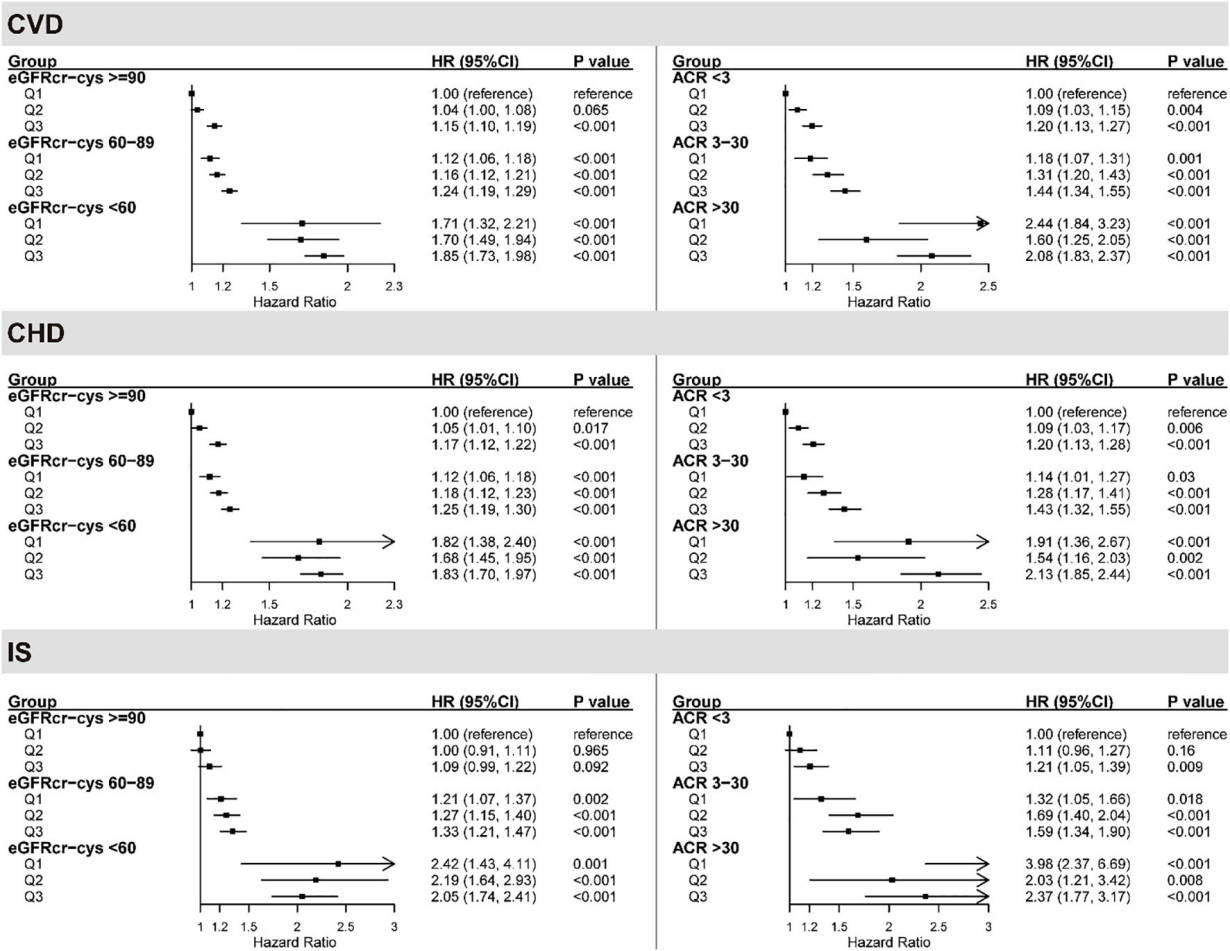
The joint association of kidney damage markers and serum urate with CVD outcomes. Multivariable model was adjusted for age, sex, income, body mass index (BMI), qualification, smoking status, alcohol status, total physical activity level, duration of sleep, fruit consumption, processed meats consumption, vegetables consumption, fishes consumption, tea consumption, coffee consumption, family history of heart diseases or stroke (only in the corresponding analysis), prevalent hypertension, prevalent diabetes, high-density lipoprotein cholesterol (HDLc), low-density lipoprotein cholesterol (LDLc), use of antihypertensive drugs, use of antihyperlipidemic drugs and use of antidiabetic drugs. The vertical line indicates the reference value of 1. ACR, albumin-to-creatinine ratio; CHD, coronary heart disease; CI, confidence interval; CVD, cardiovascular disease; eGFRcr-cys, creatinine and cystatin C-based estimated glomerular filtration rate; HR, hazard ratio; Q1, the first tertile of serum urate; Q2, the second tertile of serum urate; Q3, the third tertile of serum urate.

As shown in **Figure 2**, although no significant multiplicative interactions (*P* for interaction > 0.05) or additive interactions (**Supplementary Tables S16**) were observed between urate and eGFRcr-cys for any CVD outcome, their joint associations were evident. Participants with eGFRcr-cys < 60 mL/min/1.73 m² and the highest tertile of urate consistently exhibited the greatest risk, with a HR of 1.85 (95% CI: 1.73-1.98) for CVD compared to the reference group (eGFRcr-cys ≥ 90 and lowest urate tertile). A highly consistent risk pattern was observed for CHD and IS. The joint associations of urate with ACR showed similar results, but also in the absence of significant multiplicative interaction.

**Figure 3:**
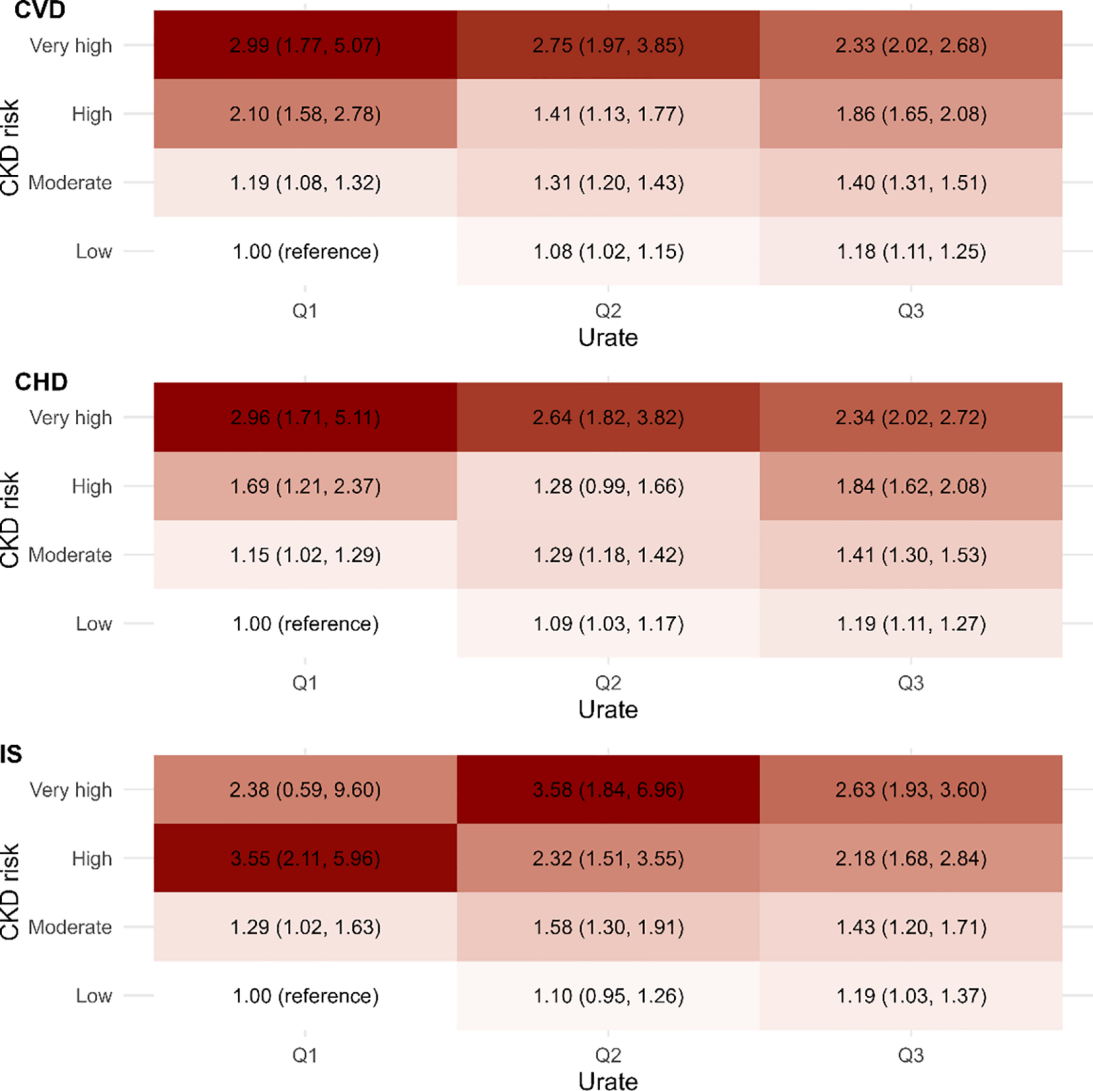
The joint association of CKD risk (defined by eGFRcr-cys and ACR) and serum urate with CVD outcomes. Multivariable model was adjusted for age, sex, income, body mass index (BMI), qualification, smoking status, alcohol status, total physical activity level, duration of sleep, fruit consumption, processed meats consumption, vegetables consumption, fishes consumption, tea consumption, coffee consumption, family history of heart diseases or stroke (only in the corresponding analysis), prevalent hypertension, prevalent diabetes, high-density lipoprotein cholesterol (HDLc), low-density lipoprotein cholesterol (LDLc), use of antihypertensive drugs, use of antihyperlipidemic drugs and use of antidiabetic drugs. The vertical line indicates the reference value of 1. ACR, albumin-to-creatinine ratio; CHD, coronary heart disease; CI, confidence interval; CVD, cardiovascular disease; eGFRcr-cys, creatinine and cystatin C-based estimated glomerular filtration rate; HR, hazard ratio; Q1, the first tertile of serum urate; Q2, the second tertile of serum urate; Q3, the third tertile of serum urate.

When we took both eGFRcr-cys and ACR into account in the assessment of renal function, it was found that participants with high CKD risk and high urate levels continued to exhibit the highest risk of CVDs (**Figure 3**). Participants at highest CKD risk and with highest tertile urate concerntration had a 2.33 times elevated risk of CVD versus participants with low CKD risk and lowest tertile of urate (HR = 2.33, 95%CI: 2.02-2.68), which was similar in CHD (HR = 2.34, 95%CI: 2.02-2.72) and IS (HR = 2.63, 95%CI: 1.93-3.60). Sensitivity analyses confirmed the robustness of these joint association findings (**Supplementary Tables S17–S21**).

### 3.6 Interaction of urate and GRS on CVD outcome risks

We examined the association between urate and GRS on CHD or IS risks in 343,054 participants of European ancestry. For CHD, the HRs (95% CIs) were 1.22 (1.18, 1.26) and 1.57 (1.51,1.63) for intermediate and high genetic risk, respectively, compared with low genetic risk. For IS, the HRs (95% CIs) were 1.14 (1.06, 1.23) and 1.27 (1.17, 1.39), respectively (**Supplementary Table S22**, **Supplementary Figure S3**).

We observed a statistically significant multiplicative interaction between serum urate and GRS for CHD (*P* for interaction < 0.05), which was consistently robust across all sensitivity analyses, including the exclusion of baseline diabetics, additional adjustment for environmental factors and diuretic use, exclusion of early incident cases, and the application of an alternative standardized GRS method (all *P* for interaction < 0.05; **Supplementary Table S23**). No significant multiplicative interaction was observed for IS (*P* for interaction = 0.529). Furthermore, assessment of additive interaction using RERI and AP revealed significant synergistic effects between elevated urate levels and high genetic risk on CHD risk (**Supplementary Table S24**). For instance, the combination of the highest urate tertile (Q3) and high genetic risk contributed to a significant relative excess risk (RERI = 0.28, 95% CI: 0.12-0.43), with 15% (AP = 0.15, 95% CI: 0.07-0.24) of the risk in this group attributable to their interaction. No such additive interaction was observed for IS.

**Figure 4:**
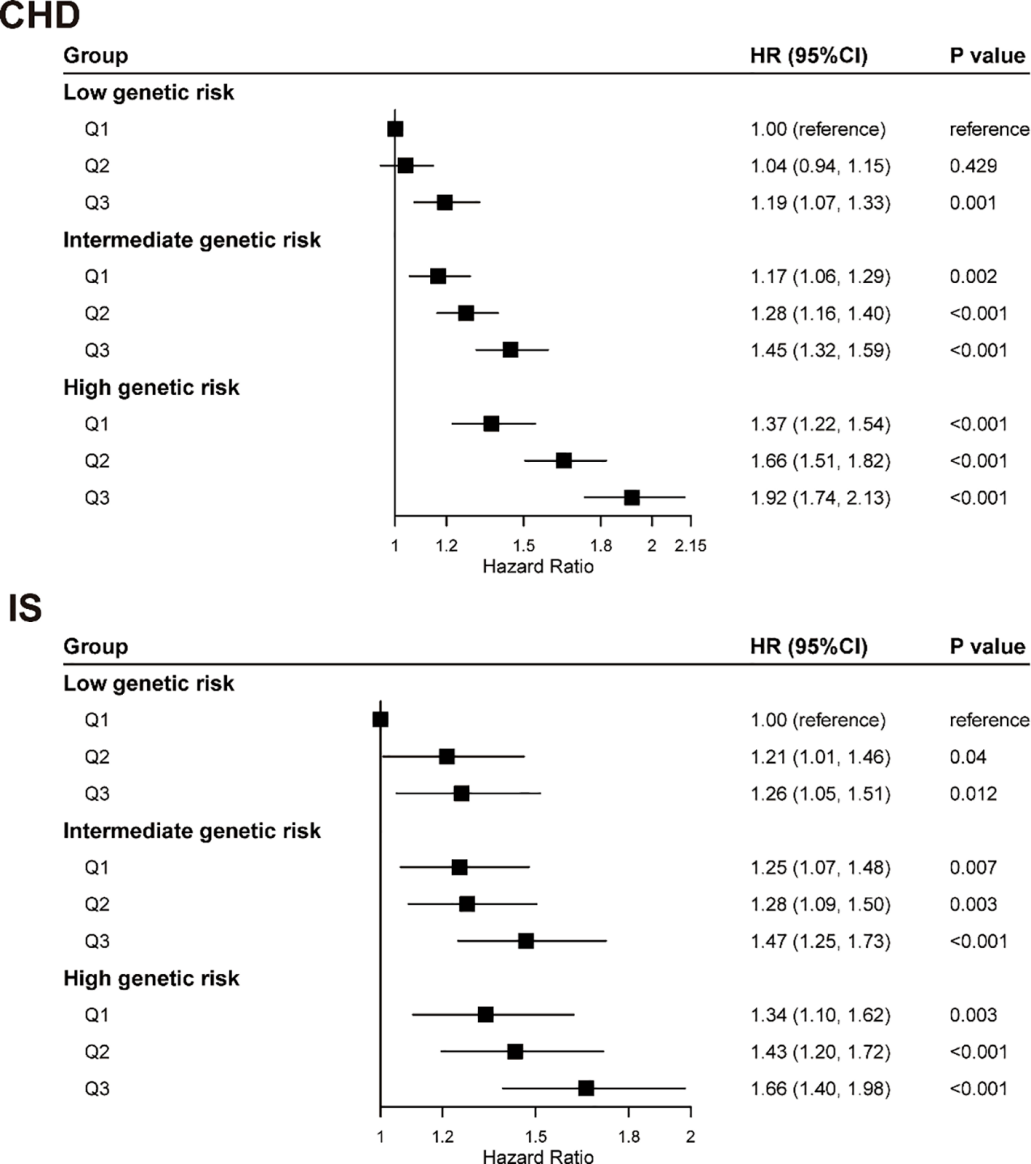
The joint association of genetic risk and serum urate with CHD and IS. There was statistically significant interaction between serum urate and genetic susceptibility to CHD (*P* for interaction = 0.0201) but no significant interaction between serum urate and genetic susceptibility to ischemic stroke (*P* for interaction = 0.5285). Multivariable model was adjusted for age, sex, income, Body mass index(BMI), qualification, smoking status, alcohol status, total physical activity level, duration of sleep, fruit consumption, processed meats consumption, vegetables consumption, fishes consumption, tea consumption, coffee consumption, family history of heart diseases or stroke (only in the corresponding analysis), prevalent hypertension, prevalent diabetes, high-density lipoprotein cholesterol (HDLc), low-density lipoprotein cholesterol (LDLc), use of antihypertensive drugs, use of antihyperlipidemic drugs and use of antidiabetic drugs. The vertical line indicates the reference value of 1. CHD, coronary heart disease; CI, confidence interval; CVD, cardiovascular disease; HR, hazard ratio; Q1, the first tertile of serum urate; Q2, the second tertile of serum urate; Q3, the third tertile of serum urate.

We further evaluated the joint associations of serum urate and genetic susceptibility (**Figure 4**). Participants with both high genetic risk and the highest tertile of serum urate exhibited the greatest risk, with a 1.92-fold increased risk of CHD (HR = 1.92, 95% CI: 1.74-2.13) compared to those with low genetic risk and the lowest urate levels. A similar pattern of joint association was observed for IS. The stability of these joint associations was confirmed in sensitivity analyses using the alternative GRS standardization method (**Supplementary Table S25**).

## 4 Discussion

In the current large prospective cohort study, we assessed the association of serum urate with CVD outcomes, and found that participants in the highest urate quintile had a 26%, 28%, and 32% higher risk of CVD, CHD, and IS, respectively, compared with participants in the lowest urate quintile. Considering the crucial importance of renal metabolism, we made further explorations of the renal function, assessed using eGFRcr-cys and ACR (separately or conjointly), and the combined influences of urate on CVDs. The results showed that participants with more severe renal decompensation and higher urate had greater risk of CVDs. Additionally, genetic risk scores for CHD and IS were further constructed and the interactions with urate were explored, identifying a stronger association between urate and CHD in individuals with higher genetic susceptibility.

In line with our findings, numerous previous studies have found a positive independent association between serum urate and the risk of CVD outcomes (4). However, there are still several other studies that have failed to demonstrate a significant association between elevated urate levels and cardiovascular events (20), which may be attributed to small sample sizes and short follow-up periods as well as inadequate control for confounding factors (21). Besides, an umbrella review of the same topic summarizing observational studies, randomized controlled trials, and Mendelian randomization studies did not found evidence for urate in in CVD (22). Building on previous studies, we used a prospective cohort design with a large sample size of a homogeneous population and controlled for potential confounders to study this issue.

It is important to integrate serum urate with renal function since they are interdependent and jointly contribute to cardiovascular health. Although we observed no statistically significant interaction between urate and impaired kidney function on CVD risk, our mediation analyses revealed that a substantial proportion of the total effect of urate on CVD outcomes was mediated by eGFRcr-cys—approximately 44% for CVD, 43% for CHD, and 52% for IS. This suggests that renal function serves as a key pathway through which urate influences cardiovascular risk. We also found that participants with healthier kidneys and lower urate would have substantially reduced risk of CVDs, which was consistent with the results of a historical cohort study (23). Importantly, Our study suggests a possible benefit of reduced cardiovascular risk from lower urate levels even in participants with severe kidney damage. Of note, we used a new race-free eGFR construction method based on blood creatinine and cystatin C in the present prospective study, because race is a social rather than a biological construct in the eGFR equation. This approach is more accurate for GFR estimation and clinical practice than other methods that have been previously reported and results in less estimation bias across race groups (14). On this basis, we included ACR indicator in accordance with the guideline (13) to more precisely assess the risk of CKD in a two-dimensional manner and to provide a more comprehensive picture of kidney health at some point.

Although the detailed mechanisms remain inconclusive, several potential pathways have been proposed to explain the association between elevated serum urate and increased risk of CVD. First, both experimental and human studies have demonstrated that increased serum urate may induce endothelial dysfunction through increased oxidative stress and inflammation (24, 25). Second, urate may play an important role in CVDs development by stimulating vascular smooth muscle cell proliferation through the vascular renin-angiotensin system (26). In addition, hyperuricemia is thought to contribute to arteriolar disease in kidney by impairing the autoregulatory response (27); and the impaired autoregulatory response of the cerebral arterioles is strongly associated with increased risk for stroke (28). Our finding of a strong mediating role of eGFRcr-cys supports the notion that renal function lies on the causal pathway between urate and CVD. It is biologically plausible that hyperuricemia and CKD-related oxidative stress and inflammation synergistically promote atherosclerosis and endothelial dysfunction (24). Nevertheless, further studies are needed to clarify the precise mechanisms underlying the joint influence of urate and renal impairment on cardiovascular outcomes.

In the current study, we observed a statistically significant multiplicative and additive interactions between urate and genetic susceptibility for CHD. The additive interaction measures provide particular insight into public health implications. We found significant positive values of both RERI and AP when comparing individuals in the highest urate tertile with high genetic risk to those in the reference group (low genetic risk and lowest urate tertile). Specifically, the RERI of 0.28 indicates substantial excess risk due to the synergistic effect between high urate and high genetic risk, while the AP of 0.15 suggests that approximately 15% of the risk in this group is attributable specifically to this interaction. These findings imply that higher genetic risk may be partially offset by maintaining lower urate levels, whereas individuals with lower genetic risk may lose their inherent protection if their urate levels become elevated. These findings are supported by a study designed to assess the interaction of genes and metabolic risk factors, which elucidated a significant interaction between *CDKAL1* polymorphisms and serum urate on the risk of type 2 diabetes (29). Since there may be an overlap in pathogenic mechanisms between type 2 diabetes and CVDs (30), this evidence partially explains our results. From a public health perspective, our results suggest that urate-lowering strategies could play an important role in the primary prevention of CHD, potentially providing greater absolute risk reduction for individuals with high genetic susceptibility. This emphasizes the value of integrating genetic risk assessment with modifiable risk factor management in precision prevention approaches.

The strengths of the current study include a large sample size and the prospective study design, which may make the association results more convincing. Of greater importance, we considered the essential role of renal functional status in these associations and assessed whether renal function would alter the association between serum urate and CVD outcomes. Another major novelty of the current study is the examination of the joint association of urate and genetic risk with CVD events. The present study also has several potential limitations. First, as an observational study, our results cannot establish causality, and residual confounding may persist despite adjustment for numerous covariates. Particularly, although we adjusted for diuretic use in sensitivity analyses, data on other specific urate-lowering medications were not fully available, and unmeasured medication effects could potentially influence the observed associations. Second, serum urate and kidney damage markers were assessed by a single measurement at baseline, which did not consider changes before and after the assessment. Future studies with repeated measurements may be helpful in addressing this issue. Third, while we restricted to European-ancestry participants to minimize population stratification in the genetic analyses, we cannot completely exclude the possibility of residual stratification. Additionally, although we selected SNPs from external GWAS that minimally overlap with UK Biobank, some degree of sample overlap remains possible, which might inflate the observed genetic effects. Fourth, this study was based on UK Biobank and most participants were of European ancestry, which might affect the generalizability of the results to other populations. Finally, despite the application of Bonferroni correction for exploratory analyses, the potential for type I error remains a common limitation in studies involving numerous statistical tests.

## 5 Conclusion

In summary, our findings from the large prospective cohort further support the association between elevated serum urate and increased risk of CVDs, and provide new insights regarding the joint effects of urate with both renal impairment and genetic susceptibility. The observed patterns of association, while requiring confirmation in additional studies, suggest that urate-lowering approaches may offer particular clinical benefit for individuals with coexisting renal dysfunction or high genetic risk. Future research incorporating repeated biomarker measurements is needed to validate these findings and further elucidate the potential causal pathways involved.

## Data Availability

Publicly available datasets were analyzed in this study. This data can be found here: https://www.ukbiobank.ac.uk/.
