# Supplementary material for: Joint impact of serum urate, renal function, and genetic susceptibility on coronary heart disease and ischemic stroke risk: a population-based study
Source: Front Endocrinol (Lausanne). 2025 Dec 12;16:1728019. doi: 10.3389/fendo.2025.1728019 (PMC12741958; doi:10.3389/fendo.2025.1728019)
Supplement: Supplementary file 1 [file Table1.docx]

**Joint Impact of Serum Urate, Renal Function, and Genetic Susceptibility on Coronary Heart Disease and Ischemic Stroke Risk: A Population-based Study**

Huangda Guo, Siyue Wang, Hexiang Peng, Tianjiao Hou, Yixin Li, Hanyu Zhang, Mengying Wang, Tao Wu, Jie Huang

**Table of contents**

[Supplementary Methods 3](#_Toc214314939)

[Calculation of the standardized weighted GRS 3](#_Toc214314940)

[Correction for multiple comparisons 3](#_Toc214314941)

[Supplemental Tables 4](#_Toc214314942)

[Table S1. Components of the genetic risk score 4](#_Toc214314943)

[Table S2. Multivariable-adjusted HRs (95%CIs) for incident cardiovascular diseases by serum urate among 383,390 participants 7](#_Toc214314944)

[Table S3. Sensitivity analyses of associations between serum urate and CVD outcomes using Fine-Gray sub-distribution hazards regression models. 8](#_Toc214314945)

[Table S4. Associations between serum urate and CVD outcomes after excluding baseline diabetic patients. 9](#_Toc214314946)

[Table S5. Associations between serum urate and CVD outcomes after additionally adjusted for environmental stressors and well-being in life. 10](#_Toc214314947)

[Table S6. Sensitivity analyses of associations between serum urate and CVD outcomes after excluding participants with incident CVDs in the previous 2 years. 11](#_Toc214314948)

[Table S7. Subgroup analyses of associations between serum urate and CVD outcomes stratified by ethnicity. 12](#_Toc214314949)

[Table S8. Sensitivity analyses of associations between serum urate and CVD outcomes additionally adjusting for diuretic use. 13](#_Toc214314950)

[Table S9. Subgroups analysis of serum urate with incident CVDs stratified by age, sex and smoking. 14](#_Toc214314951)

[Table S10. Associations between kidney damage markers and CVD outcomes after excluding baseline diabetic patients. 15](#_Toc214314952)

[Table S11. Associations between kidney damage markers and CVD outcomes after additionally adjusted for environmental stressors and well-being in life. 17](#_Toc214314953)

[Table S12. Sensitivity analyses of associations between kidney damage markers and CVD outcomes after excluding participants with incident CVDs in the previous 2 years. 19](#_Toc214314954)

[Table S13. Subgroup analyses of associations between kidney damage markers and CVD outcomes stratified by ethnicity. 21](#_Toc214314955)

[Table S14. Sensitivity analyses of associations between kidney damage markers and CVD outcomes additionally adjusting for diuretic use. 23](#_Toc214314956)

[Table S15. Mediation effect [hazard ratio (95% CI)] for renal function in the association of urate with CVDs. 25](#_Toc214314957)

[Table S16. Additive interaction between urate and kidney damage markers on CVDs risk. 26](#_Toc214314958)

[Table S17. Joint associations of urate and renal function with CVD outcomes after excluding baseline diabetic patients. 29](#_Toc214314959)

[Table S18. Joint associations of urate and renal function with CVD outcomes after additionally adjusted for environmental stressors and well-being in life. 32](#_Toc214314960)

[Table S19. Joint associations of urate and renal function with CVD outcomes after excluding participants with incident CVDs in the previous 2 years. 35](#_Toc214314961)

[Table S20. Joint associations of urate and renal function with CVD outcomes stratified by ethnicity. 38](#_Toc214314962)

[Table S22. Multivariable-adjusted HRs (95%CIs) for incident cardiovascular diseases by genetic risk among 343,054 European ancestry participants. 46](#_Toc214314963)

[Table S23. Primary analysis and sensitivity analyses of multiplicative interaction between genetic risk and serum urate with CHD. 47](#_Toc214314964)

[Table S24. Additive interaction between urate and GRSs on CVDs risk. 48](#_Toc214314965)

[Table S25. Joint associations of urate and outcome specific GRS with CVDs using standardized GRS method. 49](#_Toc214314966)

[Supplemental Figures 50](#_Toc214314967)

[Figure S1. Distribution of serum urate and polygenic risk scores. 50](#_Toc214314968)

[Figure S2. CKD risk defined by eGFR and ACR. 51](#_Toc214314969)

[Figure S3. Incident risk of cardiovascular diseases according to genetic risk among 343,054 European ancestry participants. 52](#_Toc214314970)

# Supplementary Methods

## Calculation of the standardized weighted GRS

For sensitivity analysis, an alternative genetic risk score (GRS) was calculated using a standardized weighted approach to ensure comparability and test the robustness of our findings. The formula used was:

*GRS_std_ = (Σ (βᵢ × SNPᵢ)) × N / (Σ βᵢ)*

Where:

*βᵢ* is the effect size (log odds ratio) of the i-th SNP obtained from the original GWAS publication.

*SNPᵢ* is the genotype dosage of the i-th SNP (coded as 0, 1, or 2 for the number of effect alleles).

*N* is the total number of SNPs included in the score for the specific disease (64 for CHD, 32 for IS).

*Σ βᵢ* is the sum of the effect sizes of all SNPs included in the score.

This method effectively scales the weighted sum of risk alleles, creating a score that is less dependent on the absolute magnitude of the GWAS effect sizes and facilitates interpretation across different models. The scores were then applied in the same Cox regression models as the primary GRS to test the stability of the interaction and joint effect estimates.

## Correction for multiple comparisons

To address the potential inflation of type I error due to multiple comparisons, we distinguished between hypothesis-driven and exploratory analyses. The primary associations of urate and renal function markers with cardiovascular outcomes were considered hypothesis-driven, based on substantial prior epidemiological evidence, and were thus interpreted at a nominal significance level of *P* < 0.05.

All other analyses, including the assessment of interaction terms (both multiplicative and additive) between urate and renal function markers or genetic risk scores, as well as their joint associations with outcomes, were considered exploratory. For these analyses, we applied a stringent Bonferroni correction to control the family-wise error rate. The number of independent tests in these exploratory analyses was estimated to be 11, resulting in a corrected significance threshold of *P* < 0.0045 (0.05/11). All P-values from these exploratory analyses are presented in the main text and supplementary tables and have been interpreted against this adjusted threshold. Results that reached nominal significance (*P* < 0.05) but did not survive this correction are explicitly described as “suggestive” in the text to denote a trend that requires confirmation in future studies.

# Supplemental Tables

## Table S1. Components of the genetic risk score

| SNP | Effect allele | *β* | Chr | Position | Gene | Rigion |
| --- | --- | --- | --- | --- | --- | --- |
| CHD |  |  |  |  |  |  |
| rs11206510 | T | 0.077 | 1 | 55030366 | *PCSK9* | 1p32.3 |
| rs17114036 | A | 0.122 | 1 | 56497149 | *PPAP2B* | 1p32.2 |
| rs646776 | T | 0.104 | 1 | 109275908 | *SORT1* | 1p13.3 |
| rs4845625 | T | 0.049 | 1 | 154449591 | *IL6R* | 1q21.3 |
| rs17464857 | T | 0.058 | 1 | 222589367 | *MIA3* | 1q41 |
| rs17465637 | C | 0.077 | 1 | 222650187 | *MIA3* | 1q41 |
| rs16986953 | A | 0.086 | 2 | 19742712 | *AK097927* | 2p24.1 |
| rs515135 | C | 0.068 | 2 | 21063185 | *APOB* | 2p24.1 |
| rs6544713 | T | 0.049 | 2 | 43846742 | *ABCG5-ABCG8* | 2p21 |
| rs1561198 | T | 0.058 | 2 | 85582866 | *VAMP5-VAMP8-GGCX* | 2p11.2 |
| rs2252641 | C | 0.030 | 2 | 145043894 | *ZEB2-ACO74093.1* | 2q22.3 |
| rs6725887 | C | 0.131 | 2 | 202881162 | *WDR12* | 2q33.2 |
| rs9818870 | T | 0.068 | 3 | 138403280 | *MRAS* | 3q22.3 |
| rs1878406 | T | 0.058 | 4 | 147472512 | *EDNRA* | 4q31.22 |
| rs7692387 | G | 0.068 | 4 | 155714157 | *GUCY1A3* | 4q32.1 |
| rs17087335 | T | 0.058 | 4 | 56972417 | *REST-NOA1* | 4q12 |
| rs273909 | G | 0.058 | 5 | 132331660 | *SLC22A4-SLC22A5* | 5q31.1 |
| rs6903956 | A | 0.000 | 6 | 11774350 | *ADTRP-C6orf105* | 6p24.1 |
| rs12526453 | C | 0.095 | 6 | 12927312 | *PHACTR1* | 6p24.1 |
| rs17609940 | G | 0.030 | 6 | 35067023 | *ANKS1A* | 6p21.31 |
| rs10947789 | T | 0.049 | 6 | 39207146 | *KCNK5* | 6p21.2 |
| rs12190287 | C | 0.058 | 6 | 133893387 | *TCF21* | 6q23.2 |
| rs2048327 | C | 0.058 | 6 | 160442500 | *SLC22A3-LPAL2-LPA* | 6q25.3 |
| rs4252120 | T | 0.030 | 6 | 161143608 | *PLG* | 6q14.3 |
| rs2023938 | C | 0.058 | 7 | 18997152 | *HDAC9* | 7p21.1 |
| rs10953541 | C | 0.049 | 7 | 107604100 | *DUS4L-BCAP29* | 7q22.3 |
| rs11556924 | C | 0.077 | 7 | 130023656 | *ZC3HC1* | 7q32.2 |
| rs3918226 | T | 0.131 | 7 | 150993088 | *NOS3* | 7q36.1 |
| rs264 | G | 0.058 | 8 | 19955669 | *LPL* | 8p21.3 |
| rs2954029 | A | 0.039 | 8 | 125478730 | *TRIB1* | 8q24.13 |
| rs3217992 | T | 0.131 | 9 | 22003224 | *CDKN2B-AS1,CDKN2B* | 9p21.3 |
| rs4977574 | G | 0.191 | 9 | 22098575 | *CDKN2B-AS1* | 9p21.3 |
| rs579459 | C | 0.077 | 9 | 133278724 | *ABO* | 9q34.2 |
| rs2505083 | C | 0.058 | 10 | 30046193 | *KIAA1462* | 10p11.23 |
| rs2047009 | G | 0.058 | 10 | 44539913 | *CXCL12* | 10q21.3 |
| rs501120 | T | 0.077 | 10 | 44258419 | *CXCL12* | 10q11.21 |
| rs11203042 | T | 0.039 | 10 | 89229352 | *LIPA* | 10q23.31 |
| rs1412444 | T | 0.068 | 10 | 89243170 | *LIPA* | 10q23.31 |
| rs12413409 | G | 0.077 | 10 | 102959339 | *CYP17A1-CNNM2-NT5C2* | 10q24.32 |
| rs974819 | T | 0.068 | 11 | 103789839 | *PDGFD* | 11q22.3 |
| rs964184 | G | 0.049 | 11 | 116778201 | *ZNF259-APOA5-APOA1* | 11q23.3 |
| rs10840293 | A | 0.058 | 11 | 9729649 | *SWAP70* | 11p15.4 |
| rs7136259 | T | 0.039 | 12 | 89687411 | *ATP2B1* | 12q21.33 |
| rs3184504 | T | 0.068 | 12 | 111446804 | *SH2B3* | 12q24.12 |
| rs11830157 | G | 0.039 | 12 | 117827636 | *KSR2* | 12q24.23 |
| rs9319428 | A | 0.039 | 13 | 28399484 | *FLT1* | 13q12.3 |
| rs4773144 | G | 0.049 | 13 | 110308365 | *COL4A1/A2* | 13q34 |
| rs9515203 | T | 0.068 | 13 | 110397276 | *COL4A1/A2* | 13q34 |
| rs2895811 | C | 0.039 | 14 | 99667605 | *HHIPL1* | 14q32.2 |
| rs7173743 | T | 0.077 | 15 | 78849442 | *ADAMTS7* | 15q25.1 |
| rs17514846 | A | 0.049 | 15 | 90873320 | *FURIN-FES* | 15q26.1 |
| rs56062135 | C | 0.068 | 15 | 67163292 | *SMAD3* | 15q22.33 |
| rs8042271 | G | 0.095 | 15 | 89030987 | *MFGE8-ABHD2* | 15q26.1 |
| rs216172 | C | 0.049 | 17 | 2223210 | *SMG6* | 17p13.3 |
| rs12936587 | G | 0.030 | 17 | 17640408 | *RAI1-PEMT-RASD1* | 17p11.2 |
| rs46522 | T | 0.039 | 17 | 48911235 | *UBE2Z* | 17q21.32 |
| rs7212798 | C | 0.077 | 17 | 60936127 | *BCAS3* | 17q23.2 |
| rs663129 | A | 0.058 | 18 | 60171168 | *PMAIP1-MC4R* | 18q21.32 |
| rs1122608 | G | 0.077 | 19 | 11052925 | *LDLR* | 19p13.2 |
| rs2075650 | G | 0.068 | 19 | 44892362 | *TOMM40* | 19q13.32 |
| rs445925 | G | 0.086 | 19 | 44912383 | *APOE-APOC1* | 19q13.32 |
| rs12976411 | T | -0.051 | 19 | 32391114 | *ZNF507-LOC400684* | 19q13.11 |
| rs9982601 | T | 0.113 | 21 | 34226827 | *KCNE2* | 21q22.11 |
| rs180803 | G | 0.182 | 22 | 24262890 | *POM121L9P-ADORA2A* | 22q11.23 |
| IS |  |  |  |  |  |  |
| rs880315 | C | 0.049 | 1 | 10736809 | *CASZ1* | 1p36.22 |
| rs12037987 | C | 0.068 | 1 | 112500200 | *WNT2B* | 1p13.2 |
| rs146390073 | T | 0.668 | 1 | 241142948 | *RGS7* | 1q43 |
| rs12124533 | T | 0.157 | 1 | 115115178 | *TSPAN2* | 1p13 |
| rs1052053 | G | 0.058 | 1 | 156232382 | *PMF1–SEMA4A* | 1q22 |
| rs12476527 | G | 0.049 | 2 | 26692756 | *KCNK3* | 2p23.3 |
| rs7610618 | T | 0.846 | 3 | 149439919 | *TM4SF4–TM4Sn* | 3q25.1 |
| rs34311906 | C | 0.068 | 4 | 112810934 | *ANK2* | 4q25 |
| rs17612742 | C | 0.174 | 4 | 147493499 | *EDNRA* | 4q31.22 |
| rs6825454 | C | 0.058 | 4 | 154580036 | *FGA* | 4q31.3 |
| rs13143308 | T | 0.278 | 4 | 110793263 | *PITX2* | 4q25 |
| rs11957829 | A | 0.068 | 5 | 122179500 | *LOC100505841* | 5q23.2 |
| rs6891174 | A | 0.104 | 5 | 173213587 | *NKX2-5* | 5q35.1 |
| rs16896398 | T | 0.049 | 6 | 43294966 | *SLC22A7* | 6p21.1 |
| rs4959130 | A | 0.077 | 6 | 1356681 | *FOXF2-DT* | 6p25.3 |
| rs42039 | C | 0.068 | 7 | 92615108 | *CDK6* | 7q21.2 |
| rs2107595 | A | 0.191 | 7 | 19009765 | *HDAC9, TWIST1* | 7p21.1 |
| rs7859727 | T | 0.049 | 9 | 22102166 | *CDKN2B-AS1* | 9p21.3 |
| rs10820405 | G | 0.182 | 9 | 103247955 | *LINC01492* | 9q31.1 |
| rs635634 | T | 0.077 | 9 | 133279427 | *ABO* | 9q34.2 |
| rs2295786 | A | 0.049 | 10 | 103856724 | *SH3PXD2A* | 10q24.33 |
| rs2005108 | T | 0.077 | 11 | 102899623 | *MMP12* | 11q22.2 |
| rs7304841 | A | 0.049 | 12 | 20424659 | *PDE3A* | 12p12.2 |
| rs35436 | C | 0.049 | 12 | 115116718 | *TBX3* | 12q24.21 |
| rs9526212 | G | 0.058 | 13 | 46651610 | *LRCH1* | 13q14.13 |
| rs4932370 | A | 0.049 | 15 | 90861475 | *FURIN-FES* | 15q26.1 |
| rs12932445 | C | 0.182 | 16 | 73035989 | *ZFHX3* | 16q22.3 |
| rs12445022 | A | 0.058 | 16 | 87541726 | *ZCCHC14* | 16q24.2 |
| rs11867415 | G | 0.086 | 17 | 1668524 | *PRPF8* | 17p13.3 |
| rs2229383 | T | 0.049 | 19 | 10683954 | *ILF3-SLC44A2* | 19p13.2 |
| rs8103309 | T | 0.049 | 19 | 11064259 | *SMARCA4-LDLR* | 19p13.2 |

## Table S2. Multivariable-adjusted HRs (95%CIs) for incident cardiovascular diseases by serum urate among 383,390 participants

| **Serum urate** | **HR (95%CI)** | ***P*** | ***P* for trend** |
| --- | --- | --- | --- |
| **CVD** |  |  | <0.001 |
| Quintile 1 | 1.00 (reference) | reference |  |
| Quintile 2 | 1.04(1.00,1.08) | 0.066 |  |
| Quintile 3 | **1.09(1.04,1.13)** | <0.001 |  |
| Quintile 4 | **1.14(1.09,1.19)** | <0.001 |  |
| Quintile 5 | **1.26(1.21,1.31)** | <0.001 |  |
| **CHD** |  |  | <0.001 |
| Quintile 1 | 1.00 (reference) | reference |  |
| Quintile 2 | 1.04(1.00,1.09) | 0.064 |  |
| Quintile 3 | **1.11(1.06,1.16)** | <0.001 |  |
| Quintile 4 | **1.15(1.10,1.21)** | <0.001 |  |
| Quintile 5 | **1.28(1.22,1.34)** | <0.001 |  |
| **Ischemic Stroke** |  |  | <0.001 |
| Quintile 1 | 1.00 (reference) | reference |  |
| Quintile 2 | 1.10(0.99,1.22) | 0.076 |  |
| Quintile 3 | **1.12(1.01,1.24)** | 0.033 |  |
| Quintile 4 | **1.19(1.07,1.32)** | 0.001 |  |
| Quintile 5 | **1.32(1.19,1.47)** | <0.001 |  |

CVD, cardiovascular disease; CHD, coronary heart disease; CI, confidence interval; HR, hazard ratio.

Multivariable model was adjusted for age, sex, income score, ethnic, Body mass index(BMI), qualification, smoking status, alcohol status, total physical activity level, duration of sleep, fruit consumption, processed meats consumption, vegetables consumption, fishes consumption, tea consumption, coffee consumption, family history of heart diseases or stroke (only in the corresponding analysis), prevalent hypertension, prevalent diabetes, high-density lipoprotein cholesterol (HDLc), low-density lipoprotein cholesterol (LDLc), use of antihypertensive drugs, use of antihyperlipidemic drugs and use of antidiabetic drugs. Significant results are indicated in bold (*P* < 0.05).

## Table S3. Sensitivity analyses of associations between serum urate and CVD outcomes using Fine-Gray sub-distribution hazards regression models.

| Urate | CVD | | CHD | | IS | |
| --- | --- | --- | --- | --- | --- | --- |
|  | HR (95% CI) | *P* | HR (95% CI) | *P* | HR (95% CI) | *P* |
| Quintile 1 | 1.00 (reference) | reference | 1.00 (reference) | reference | 1.00 (reference) | reference |
| Quintile 2 | 1.04 (1.00, 1.08) | 0.066 | 1.04 (1.00, 1.09) | 0.064 | 1.10 (0.99, 1.22) | 0.077 |
| Quintile 3 | 1.09 (1.04, 1.13) | <0.001 | 1.11 (1.06, 1.16) | <0.001 | 1.12 (1.01, 1.24) | 0.036 |
| Quintile 4 | 1.14 (1.09, 1.19) | <0.001 | 1.15 (1.10, 1.21) | <0.001 | 1.19 (1.07, 1.32) | 0.001 |
| Quintile 5 | 1.26 (1.20, 1.31) | <0.001 | 1.28 (1.22, 1.34) | <0.001 | 1.32 (1.18, 1.47) | <0.001 |

CHD, coronary heart disease; CI, confidence interval; CVD, cardiovascular disease; HR, hazard ratio, IS, ischemic stroke.

Multivariable model was adjusted for age, sex, income score, ethnic, Body mass index(BMI), qualification, smoking status, alcohol status, total physical activity level, duration of sleep, fruit consumption, processed meats consumption, vegetables consumption, fishes consumption, tea consumption, coffee consumption, family history of heart diseases or stroke (only in the corresponding analysis), prevalent hypertension, prevalent diabetes, high-density lipoprotein cholesterol (HDLc), low-density lipoprotein cholesterol (LDLc), use of antihypertensive drugs, use of antihyperlipidemic drugs and use of antidiabetic drugs.

## Table S4. Associations between serum urate and CVD outcomes after excluding baseline diabetic patients.

| Urate | CVD | | CHD | | IS | |
| --- | --- | --- | --- | --- | --- | --- |
|  | HR (95% CI) | *P* | HR (95% CI) | *P* | HR (95% CI) | *P* |
| Quintile 1 | 1.00 (reference) | reference | 1.00 (reference) | reference | 1.00 (reference) | reference |
| Quintile 2 | 1.04 (1.00, 1.09) | 0.053 | 1.05 (1.00, 1.10) | 0.075 | 1.13 (1.01, 1.26) | 0.033 |
| Quintile 3 | 1.10 (1.06, 1.15) | <0.001 | 1.12 (1.07, 1.18) | <0.001 | 1.16 (1.04, 1.30) | 0.010 |
| Quintile 4 | 1.14 (1.09, 1.20) | <0.001 | 1.15 (1.09, 1.21) | <0.001 | 1.24 (1.10, 1.39) | <0.001 |
| Quintile 5 | 1.26 (1.20, 1.32) | <0.001 | 1.28 (1.21, 1.35) | <0.001 | 1.34 (1.19, 1.51) | <0.001 |

CHD, coronary heart disease; CI, confidence interval; CVD, cardiovascular disease; HR, hazard ratio, IS, ischemic stroke.

Multivariable model was adjusted for age, sex, income score, ethnic, Body mass index(BMI), qualification, smoking status, alcohol status, total physical activity level, duration of sleep, fruit consumption, processed meats consumption, vegetables consumption, fishes consumption, tea consumption, coffee consumption, family history of heart diseases or stroke (only in the corresponding analysis), prevalent hypertension, high-density lipoprotein cholesterol (HDLc), low-density lipoprotein cholesterol (LDLc), use of antihypertensive drugs, use of antihyperlipidemic drugs and use of antidiabetic drugs.

## Table S5. Associations between serum urate and CVD outcomes after additionally adjusted for environmental stressors and well-being in life.

| Urate | CVD | | CHD | | IS | |
| --- | --- | --- | --- | --- | --- | --- |
|  | HR (95% CI) | *P* | HR (95% CI) | *P* | HR (95% CI) | *P* |
| Quintile 1 | 1.00 (reference) | reference | 1.00 (reference) | reference | 1.00 (reference) | reference |
| Quintile 2 | 1.04 (1.00, 1.08) | 0.060 | 1.05 (1.00, 1.10) | 0.058 | 1.10 (0.99, 1.22) | 0.075 |
| Quintile 3 | 1.09 (1.05, 1.13) | <0.001 | 1.11 (1.06, 1.16) | <0.001 | 1.12 (1.01, 1.24) | 0.033 |
| Quintile 4 | 1.14 (1.10, 1.19) | <0.001 | 1.15 (1.10, 1.21) | <0.001 | 1.19 (1.07, 1.32) | <0.001 |
| Quintile 5 | 1.26 (1.21, 1.31) | <0.001 | 1.28 (1.22, 1.34) | <0.001 | 1.32 (1.19, 1.47) | <0.001 |

CHD, coronary heart disease; CI, confidence interval; CVD, cardiovascular disease; HR, hazard ratio, IS, ischemic stroke.

Multivariable model was adjusted for age, sex, income score, ethnic, Body mass index(BMI), qualification, smoking status, alcohol status, total physical activity level, duration of sleep, fruit consumption, processed meats consumption, vegetables consumption, fishes consumption, tea consumption, coffee consumption, family history of heart diseases or stroke (only in the corresponding analysis), prevalent hypertension, prevalent diabetes, high-density lipoprotein cholesterol (HDLc), low-density lipoprotein cholesterol (LDLc), use of antihypertensive drugs, use of antihyperlipidemic drugs and use of antidiabetic drugs, residential noise pollution, PM10, and well-being in life.

## Table S6. Sensitivity analyses of associations between serum urate and CVD outcomes after excluding participants with incident CVDs in the previous 2 years.

| Urate | CVD | | CHD | | IS | |
| --- | --- | --- | --- | --- | --- | --- |
|  | HR (95% CI) | *P* | HR (95% CI) | *P* | HR (95% CI) | *P* |
| Quintile 1 | 1.00 (reference) | reference | 1.00 (reference) | reference | 1.00 (reference) | reference |
| Quintile 2 | 1.04 (1.00, 1.09) | 0.075 | 1.04 (0.99, 1.09) | 0.104 | 1.08 (0.97, 1.21) | 0.143 |
| Quintile 3 | 1.09 (1.05, 1.14) | <0.001 | 1.11 (1.06, 1.16) | <0.001 | 1.13 (1.02, 1.26) | 0.024 |
| Quintile 4 | 1.14 (1.09, 1.19) | <0.001 | 1.14 (1.09, 1.20) | <0.001 | 1.19 (1.07, 1.33) | 0.002 |
| Quintile 5 | 1.24 (1.19, 1.30) | <0.001 | 1.26 (1.20, 1.32) | <0.001 | 1.31 (1.17, 1.46) | <0.001 |

CHD, coronary heart disease; CI, confidence interval; CVD, cardiovascular disease; HR, hazard ratio, IS, ischemic stroke.

Multivariable model was adjusted for age, sex, income score, ethnic, Body mass index(BMI), qualification, smoking status, alcohol status, total physical activity level, duration of sleep, fruit consumption, processed meats consumption, vegetables consumption, fishes consumption, tea consumption, coffee consumption, family history of heart diseases or stroke (only in the corresponding analysis), prevalent hypertension, prevalent diabetes, high-density lipoprotein cholesterol (HDLc), low-density lipoprotein cholesterol (LDLc), use of antihypertensive drugs, use of antihyperlipidemic drugs and use of antidiabetic drugs.

## Table S7. Subgroup analyses of associations between serum urate and CVD outcomes stratified by ethnicity.

| Ethnic | Urate | CVD | | CHD | | IS | |
| --- | --- | --- | --- | --- | --- | --- | --- |
|  |  | HR (95% CI) | *P* | HR (95% CI) | *P* | HR (95% CI) | *P* |
| White | Quintile 1 | 1.00 (reference) | reference | 1.00 (reference) | reference | 1.00 (reference) | reference |
|  | Quintile 2 | 1.04 (1.00, 1.09) | 0.062 | 1.05 (1.00, 1.10) | 0.062 | 1.10 (0.98, 1.22) | 0.098 |
|  | Quintile 3 | 1.10 (1.06, 1.15) | <0.001 | 1.13 (1.08, 1.19) | <0.001 | 1.12 (1.00, 1.24) | 0.047 |
|  | Quintile 4 | 1.15 (1.11, 1.21) | <0.001 | 1.17 (1.11, 1.23) | <0.001 | 1.20 (1.07, 1.33) | 0.001 |
|  | Quintile 5 | 1.27 (1.21, 1.33) | <0.001 | 1.30 (1.23, 1.36) | <0.001 | 1.32 (1.19, 1.48) | <0.001 |
| Other | Quintile 1 | 1.00 (reference) | reference | 1.00 (reference) | reference | 1.00 (reference) | reference |
|  | Quintile 2 | 1.01 (0.88, 1.16) | 0.894 | 1.01 (0.87, 1.18) | 0.857 | 1.13 (0.79, 1.62) | 0.513 |
|  | Quintile 3 | 0.94 (0.82, 1.08) | 0.364 | 0.92 (0.79, 1.07) | 0.267 | 1.12 (0.79, 1.61) | 0.52 |
|  | Quintile 4 | 1.00 (0.87, 1.15) | 0.968 | 0.99 (0.85, 1.15) | 0.887 | 1.11 (0.77, 1.60) | 0.583 |
|  | Quintile 5 | 1.14 (0.99, 1.31) | 0.067 | 1.13 (0.96, 1.31) | 0.132 | 1.25 (0.87, 1.81) | 0.231 |

CHD, coronary heart disease; CI, confidence interval; CVD, cardiovascular disease; HR, hazard ratio, IS, ischemic stroke.

Multivariable model was adjusted for age, sex, income score, Body mass index(BMI), qualification, smoking status, alcohol status, total physical activity level, duration of sleep, fruit consumption, processed meats consumption, vegetables consumption, fishes consumption, tea consumption, coffee consumption, family history of heart diseases or stroke (only in the corresponding analysis), prevalent hypertension, prevalent diabetes, high-density lipoprotein cholesterol (HDLc), low-density lipoprotein cholesterol (LDLc), use of antihypertensive drugs, use of antihyperlipidemic drugs and use of antidiabetic drugs.

## Table S8. Sensitivity analyses of associations between serum urate and CVD outcomes additionally adjusting for diuretic use.

| Urate | CVD | | CHD | | IS | |
| --- | --- | --- | --- | --- | --- | --- |
|  | HR (95% CI) | *P* | HR (95% CI) | *P* | HR (95% CI) | *P* |
| Quintile 1 | 1.00 (reference) | reference | 1.00 (reference) | reference | 1.00 (reference) | reference |
| Quintile 2 | 1.04 (1.00, 1.08) | 0.065 | 1.05 (1.00, 1.09) | 0.061 | 1.10 (0.99, 1.22) | 0.077 |
| Quintile 3 | 1.09 (1.04, 1.13) | <0.001 | 1.11 (1.06, 1.16) | <0.001 | 1.12 (1.01, 1.24) | 0.036 |
| Quintile 4 | 1.14 (1.09, 1.18) | <0.001 | 1.15 (1.09, 1.20) | <0.001 | 1.19 (1.07, 1.32) | 0.001 |
| Quintile 5 | 1.24 (1.19, 1.29) | <0.001 | 1.26 (1.20, 1.32) | <0.001 | 1.30 (1.17, 1.45) | <0.001 |

CHD, coronary heart disease; CI, confidence interval; CVD, cardiovascular disease; HR, hazard ratio, IS, ischemic stroke.

Multivariable model was adjusted for age, sex, income score, ethnic, Body mass index(BMI), qualification, smoking status, alcohol status, total physical activity level, duration of sleep, fruit consumption, processed meats consumption, vegetables consumption, fishes consumption, tea consumption, coffee consumption, family history of heart diseases or stroke (only in the corresponding analysis), prevalent hypertension, prevalent diabetes, high-density lipoprotein cholesterol (HDLc), low-density lipoprotein cholesterol (LDLc), use of antihypertensive drugs, use of antihyperlipidemic drugs and use of antidiabetic drugs, diuretic use.

## Table S9. Subgroups analysis of serum urate with incident CVDs stratified by age, sex and smoking.

|  | CVD |  | CHD |  | IS |  |
| --- | --- | --- | --- | --- | --- | --- |
|  | HR (95% CI) | *P* for interaction | HR (95% CI) | *P* for interaction | HR (95% CI) | *P* for interaction |
| Total | **1.09 (1.08,1.11)** | | **1.09 (1.08,1.11)** | | **1.12 (1.08,1.15)** | |
| Age |  | <0.001 |  | <0.001 |  | <0.001 |
| <60 y | **1.09 (1.07,1.10)** | | **1.09 (1.07,1.10)** | | **1.11 (1.07,1.16)** | |
| ≥60 y | **1.12 (1.10,1.14)** | | **1.12 (1.10,1.15)** | | **1.17 (1.11,1.24)** | |
| Sex |  | <0.001 |  | <0.001 |  | 0.001 |
| Women | **1.10 (1.08,1.13)** | | **1.11 (1.08,1.14)** | | **1.15 (1.09,1.21)** | |
| Men | **1.06 (1.04,1.08)** | | **1.06 (1.04,1.07)** | | **1.08 (1.04,1.12)** | |
| Smoking status | | <0.001 |  | <0.001 |  | <0.001 |
| Non-current | **1.06 (1.03,1.10)** | | **1.07 (1.03,1.11)** | | 1.03 (0.95,1.12) | |
| Current | **1.10 (1.09,1.11)** | | **1.10 (1.09,1.12)** | | **1.14 (1.10,1.17)** | |

CHD, coronary heart disease; CI, confidence interval; CVD, cardiovascular disease; HR, hazard ratio; IS, ischemic stroke.

We analyzed the association of one standard deviation elevation in serum urate with the risk of CVD, CHD, and IS in different subgroups. Significant results are indicated in bold (*P* < 0.05).

## Table S10. Associations between kidney damage markers and CVD outcomes after excluding baseline diabetic patients.

| Group | CVD | | CHD | | IS | |
| --- | --- | --- | --- | --- | --- | --- |
|  | HR (95% CI) | *P* | HR (95% CI) | *P* | HR (95% CI) | *P* |
| eGFRcr-cys, mL/min/1.73 m^2^ | | |  |  |  |  |
| G1 (≥90) | 1.00 (reference) | reference | 1.00 (reference) | reference | 1.00 (reference) | reference |
| G2 (60-89) | 1.12 (1.09, 1.14) | <0.001 | 1.10 (1.07, 1.13) | <0.001 | 1.23 (1.16, 1.31) | <0.001 |
| G3a (45-59) | 1.48 (1.37, 1.60) | <0.001 | 1.40 (1.28, 1.52) | <0.001 | 1.94 (1.63, 2.32) | <0.001 |
| G3b (30-44) | 1.99 (1.71, 2.33) | <0.001 | 2.02 (1.72, 2.38) | <0.001 | 2.15 (1.47, 3.16) | <0.001 |
| G4 (15-29) | 2.61 (1.99, 3.42) | <0.001 | 2.40 (1.79, 3.23) | <0.001 | 2.96 (1.54, 5.72) | 0.001 |
| G5 (<15) | 2.26 (1.36, 3.76) | 0.002 | 2.48 (1.49, 4.11) | <0.001 | 1.22 (0.17, 8.67) | 0.843 |
| ACR, mg/mmol | |  |  |  |  |  |
| A1 (<3) | 1.00 (reference) | reference | 1.00 (reference) | reference | 1.00 (reference) | reference |
| A2 (3-30) | 1.17 (1.11, 1.23) | <0.001 | 1.14 (1.08, 1.21) | <0.001 | 1.31 (1.17, 1.47) | <0.001 |
| A3 (>30) | 1.54 (1.34, 1.77) | <0.001 | 1.52 (1.30, 1.77) | <0.001 | 1.67 (1.20, 2.31) | 0.002 |
| eGFRcr-cys & ACR | |  |  |  |  |  |
| Low | 1.00 (reference) | reference | 1.00 (reference) | reference | 1.00 (reference) | reference |
| Moderate | 1.18 (1.12, 1.24) | <0.001 | 1.16 (1.09, 1.22) | <0.001 | 1.25 (1.12, 1.41) | <0.001 |
| High | 1.48 (1.31, 1.67) | <0.001 | 1.36 (1.19, 1.56) | <0.001 | 1.85 (1.42, 2.40) | <0.001 |
| Very high | 1.80 (1.53, 2.12) | <0.001 | 1.83 (1.54, 2.18) | <0.001 | 1.96 (1.33, 2.87) | <0.001 |

CHD, coronary heart disease; CI, confidence interval; CVD, cardiovascular disease; HR, hazard ratio, IS, ischemic stroke.

Multivariable model was adjusted for age, sex, income score, ethnic, Body mass index(BMI), qualification, smoking status, alcohol status, total physical activity level, duration of sleep, fruit consumption, processed meats consumption, vegetables consumption, fishes consumption, tea consumption, coffee consumption, family history of heart diseases or stroke (only in the corresponding analysis), prevalent hypertension, high-density lipoprotein cholesterol (HDLc), low-density lipoprotein cholesterol (LDLc), use of antihypertensive drugs, use of antihyperlipidemic drugs and use of antidiabetic drugs.

## Table S11. Associations between kidney damage markers and CVD outcomes after additionally adjusted for environmental stressors and well-being in life.

| Group | CVD | | CHD | | IS | |
| --- | --- | --- | --- | --- | --- | --- |
|  | HR (95% CI) | *P* | HR (95% CI) | *P* | HR (95% CI) | *P* |
| eGFRcr-cys, mL/min/1.73 m^2^ | | |  |  |  |  |
| G1 (≥90) | 1.00 (reference) | reference | 1.00 (reference) | reference | 1.00 (reference) | reference |
| G2 (60-89) | 1.12 (1.10, 1.15) | <0.001 | 1.11 (1.09, 1.14) | <0.001 | 1.25 (1.18, 1.33) | <0.001 |
| G3a (45-59) | 1.54 (1.44, 1.64) | <0.001 | 1.50 (1.39, 1.61) | <0.001 | 1.87 (1.60, 2.17) | <0.001 |
| G3b (30-44) | 2.08 (1.85, 2.34) | <0.001 | 2.07 (1.83, 2.35) | <0.001 | 2.19 (1.65, 2.92) | <0.001 |
| G4 (15-29) | 2.63 (2.13, 3.23) | <0.001 | 2.36 (1.88, 2.97) | <0.001 | 3.63 (2.33, 5.65) | <0.001 |
| G5 (<15) | 3.63 (2.57, 5.14) | <0.001 | 4.05 (2.86, 5.74) | <0.001 | 3.20 (1.33, 7.70) | 0.010 |
| ACR, mg/mmol | |  |  |  |  |  |
| A1 (<3) | 1.00 (reference) | reference | 1.00 (reference) | reference | 1.00 (reference) | reference |
| A2 (3-30) | 1.20 (1.15, 1.26) | <0.001 | 1.18 (1.12, 1.23) | <0.001 | 1.39 (1.25, 1.53) | <0.001 |
| A3 (>30) | 1.79 (1.62, 1.98) | <0.001 | 1.75 (1.57, 1.96) | <0.001 | 2.17 (1.74, 2.70) | <0.001 |
| eGFRcr-cys & ACR | |  |  |  |  |  |
| Low | 1.00 (reference) | reference | 1.00 (reference) | reference | 1.00 (reference) | reference |
| Moderate | 1.20 (1.15, 1.26) | <0.001 | 1.18 (1.13, 1.24) | <0.001 | 1.30 (1.17, 1.43) | <0.001 |
| High | 1.60 (1.46, 1.75) | <0.001 | 1.52 (1.38, 1.68) | <0.001 | 2.07 (1.70, 2.52) | <0.001 |
| Very high | 2.13 (1.89, 2.41) | <0.001 | 2.12 (1.86, 2.41) | <0.001 | 2.40 (1.84, 3.13) | <0.001 |

CHD, coronary heart disease; CI, confidence interval; CVD, cardiovascular disease; HR, hazard ratio, IS, ischemic stroke.

Multivariable model was adjusted for age, sex, income score, ethnic, Body mass index(BMI), qualification, smoking status, alcohol status, total physical activity level, duration of sleep, fruit consumption, processed meats consumption, vegetables consumption, fishes consumption, tea consumption, coffee consumption, family history of heart diseases or stroke (only in the corresponding analysis), prevalent hypertension, prevalent diabetes, high-density lipoprotein cholesterol (HDLc), low-density lipoprotein cholesterol (LDLc), use of antihypertensive drugs, use of antihyperlipidemic drugs and use of antidiabetic drugs, residential noise pollution, PM10, and well-being in life.

## Table S12. Sensitivity analyses of associations between kidney damage markers and CVD outcomes after excluding participants with incident CVDs in the previous 2 years.

| Group | CVD | | CHD | | IS | |
| --- | --- | --- | --- | --- | --- | --- |
|  | HR (95% CI) | *P* | HR (95% CI) | *P* | HR (95% CI) | *P* |
| eGFRcr-cys, mL/min/1.73 m^2^ | | |  |  |  |  |
| G1 (≥90) | 1.00 (reference) | reference | 1.00 (reference) | reference | 1.00 (reference) | reference |
| G2 (60-89) | 1.12 (1.09, 1.14) | <0.001 | 1.11 (1.08, 1.14) | <0.001 | 1.22 (1.15, 1.30) | <0.001 |
| G3a (45-59) | 1.54 (1.43, 1.65) | <0.001 | 1.48 (1.38, 1.60) | <0.001 | 1.86 (1.59, 2.18) | <0.001 |
| G3b (30-44) | 2.11 (1.86, 2.39) | <0.001 | 2.10 (1.84, 2.40) | <0.001 | 2.24 (1.67, 3.01) | <0.001 |
| G4 (15-29) | 2.72 (2.17, 3.40) | <0.001 | 2.40 (1.87, 3.08) | <0.001 | 3.79 (2.41, 5.97) | <0.001 |
| G5 (<15) | 3.03 (2.00, 4.61) | <0.001 | 3.40 (2.23, 5.16) | <0.001 | 2.11 (0.68, 6.55) | 0.198 |
| ACR, mg/mmol | |  |  |  |  |  |
| A1 (<3) | 1.00 (reference) | reference | 1.00 (reference) | reference | 1.00 (reference) | reference |
| A2 (3-30) | 1.18 (1.13, 1.24) | <0.001 | 1.16 (1.10, 1.22) | <0.001 | 1.35 (1.22, 1.50) | <0.001 |
| A3 (>30) | 1.73 (1.54, 1.93) | <0.001 | 1.68 (1.48, 1.89) | <0.001 | 2.17 (1.73, 2.73) | <0.001 |
| eGFRcr-cys & ACR | |  |  |  |  |  |
| Low | 1.00 (reference) | reference | 1.00 (reference) | reference | 1.00 (reference) | reference |
| Moderate | 1.18 (1.12, 1.23) | <0.001 | 1.16 (1.10, 1.22) | <0.001 | 1.27 (1.14, 1.41) | <0.001 |
| High | 1.60 (1.45, 1.77) | <0.001 | 1.53 (1.37, 1.70) | <0.001 | 2.08 (1.70, 2.55) | <0.001 |
| Very high | 2.11 (1.85, 2.41) | <0.001 | 2.06 (1.79, 2.37) | <0.001 | 2.44 (1.85, 3.21) | <0.001 |

CHD, coronary heart disease; CI, confidence interval; CVD, cardiovascular disease; HR, hazard ratio, IS, ischemic stroke.

Multivariable model was adjusted for age, sex, income score, ethnic, Body mass index(BMI), qualification, smoking status, alcohol status, total physical activity level, duration of sleep, fruit consumption, processed meats consumption, vegetables consumption, fishes consumption, tea consumption, coffee consumption, family history of heart diseases or stroke (only in the corresponding analysis), prevalent hypertension, prevalent diabetes, high-density lipoprotein cholesterol (HDLc), low-density lipoprotein cholesterol (LDLc), use of antihypertensive drugs, use of antihyperlipidemic drugs and use of antidiabetic drugs.

## Table S13. Subgroup analyses of associations between kidney damage markers and CVD outcomes stratified by ethnicity.

| Ethnic | Group | CVD | | CHD | | IS | |
| --- | --- | --- | --- | --- | --- | --- | --- |
|  |  | HR (95% CI) | *P* | HR (95% CI) | *P* | HR (95% CI) | *P* |
| eGFRcr-cys, mL/min/1.73 m^2^ | | |  |  |  |  |  |
| White | G1 (≥90) | 1.00 (reference) | reference | 1.00 (reference) | reference | 1.00 (reference) | reference |
|  | G2 (60-89) | 1.12 (1.10, 1.15) | <0.001 | 1.11 (1.08, 1.14) | <0.001 | 1.27 (1.19, 1.35) | <0.001 |
|  | G3a (45-59) | 1.53 (1.43, 1.64) | <0.001 | 1.48 (1.38, 1.60) | <0.001 | 1.92 (1.65, 2.25) | <0.001 |
|  | G3b (30-44) | 2.18 (1.94, 2.46) | <0.001 | 2.16 (1.90, 2.46) | <0.001 | 2.44 (1.83, 3.25) | <0.001 |
|  | G4 (15-29) | 2.42 (1.93, 3.03) | <0.001 | 2.23 (1.74, 2.86) | <0.001 | 3.20 (1.95, 5.24) | <0.001 |
|  | G5 (<15) | 3.73 (2.54, 5.48) | <0.001 | 4.07 (2.77, 5.98) | <0.001 | 3.41 (1.28, 9.12) | 0.014 |
| Other | G1 (≥90) | 1.00 (reference) | reference | 1.00 (reference) | reference | 1.00 (reference) | reference |
|  | G2 (60-89) | 1.13 (1.04, 1.22) | 0.003 | 1.12 (1.03, 1.22) | 0.01 | 1.09 (0.89, 1.34) | 0.423 |
|  | G3a (45-59) | 1.68 (1.33, 2.12) | <0.001 | 1.71 (1.34, 2.19) | <0.001 | 1.29 (0.69, 2.41) | 0.419 |
|  | G3b (30-44) | 1.10 (0.66, 1.84) | 0.715 | 1.23 (0.73, 2.05) | 0.44 | 0.00 (0.00, Inf) | 0.986 |
|  | G4 (15-29) | 5.30 (3.12, 9.00) | <0.001 | 3.39 (1.91, 6.03) | <0.001 | 10.40 (3.82, 28.31) | <0.001 |
|  | G5 (<15) | 3.52 (1.57, 7.89) | 0.002 | 4.35 (1.94, 9.78) | <0.001 | 2.40 (0.33, 17.40) | 0.387 |
| ACR, mg/mmol | |  |  |  |  |  |  |
| White | A1 (<3) | 1.00 (reference) | reference | 1.00 (reference) | reference | 1.00 (reference) | reference |
|  | A2 (3-30) | 1.20 (1.14, 1.25) | <0.001 | 1.17 (1.12, 1.23) | <0.001 | 1.37 (1.24, 1.53) | <0.001 |
|  | A3 (>30) | 1.78 (1.59, 1.99) | <0.001 | 1.74 (1.54, 1.96) | <0.001 | 2.15 (1.69, 2.72) | <0.001 |
| Other | A1 (<3) | 1.00 (reference) | reference | 1.00 (reference) | reference | 1.00 (reference) | reference |
|  | A2 (3-30) | 1.24 (1.08, 1.41) | 0.002 | 1.20 (1.04, 1.39) | 0.013 | 1.50 (1.09, 2.05) | 0.012 |
|  | A3 (>30) | 1.87 (1.43, 2.46) | <0.001 | 1.84 (1.37, 2.47) | <0.001 | 2.38 (1.33, 4.28) | 0.004 |
| eGFRcr-cys & ACR | |  |  |  |  |  |  |
| White | Low | 1.00 (reference) | reference | 1.00 (reference) | reference | 1.00 (reference) | reference |
|  | Moderate | 1.20 (1.14, 1.25) | <0.001 | 1.18 (1.12, 1.24) | <0.001 | 1.27 (1.15, 1.42) | <0.001 |
|  | High | 1.59 (1.45, 1.76) | <0.001 | 1.51 (1.36, 1.68) | <0.001 | 2.10 (1.71, 2.58) | <0.001 |
|  | Very high | 2.14 (1.88, 2.43) | <0.001 | 2.14 (1.86, 2.45) | <0.001 | 2.39 (1.80, 3.17) | <0.001 |
| Other | Low | 1.00 (reference) | reference | 1.00 (reference) | reference | 1.00 (reference) | reference |
|  | Moderate | 1.24 (1.09, 1.42) | 0.001 | 1.20 (1.04, 1.39) | 0.015 | 1.51 (1.10, 2.07) | 0.011 |
|  | High | 1.70 (1.29, 2.24) | <0.001 | 1.66 (1.23, 2.23) | <0.001 | 2.04 (1.09, 3.80) | 0.025 |
|  | Very high | 2.13 (1.50, 3.03) | <0.001 | 2.01 (1.38, 2.92) | <0.001 | 2.88 (1.37, 6.02) | 0.005 |

CHD, coronary heart disease; CI, confidence interval; CVD, cardiovascular disease; HR, hazard ratio, IS, ischemic stroke.

Multivariable model was adjusted for age, sex, income score, Body mass index(BMI), qualification, smoking status, alcohol status, total physical activity level, duration of sleep, fruit consumption, processed meats consumption, vegetables consumption, fishes consumption, tea consumption, coffee consumption, family history of heart diseases or stroke (only in the corresponding analysis), prevalent hypertension, prevalent diabetes, high-density lipoprotein cholesterol (HDLc), low-density lipoprotein cholesterol (LDLc), use of antihypertensive drugs, use of antihyperlipidemic drugs and use of antidiabetic drugs.

## Table S14. Sensitivity analyses of associations between kidney damage markers and CVD outcomes additionally adjusting for diuretic use.

| Group | CVD | | CHD | | IS | |
| --- | --- | --- | --- | --- | --- | --- |
|  | HR (95% CI) | *P* | HR (95% CI) | *P* | HR (95% CI) | *P* |
| eGFRcr-cys, mL/min/1.73 m^2^ | | |  |  |  |  |
| G1 (≥90) | 1.00 (reference) | reference | 1.00 (reference) | reference | 1.00 (reference) | reference |
| G2 (60-89) | 1.12 (1.09, 1.14) | <0.001 | 1.11 (1.08, 1.13) | <0.001 | 1.25 (1.17, 1.32) | <0.001 |
| G3a (45-59) | 1.46 (1.37, 1.56) | <0.001 | 1.41 (1.31, 1.51) | <0.001 | 1.81 (1.55, 2.11) | <0.001 |
| G3b (30-44) | 1.85 (1.65, 2.09) | <0.001 | 1.83 (1.62, 2.07) | <0.001 | 2.03 (1.52, 2.71) | <0.001 |
| G4 (15-29) | 2.14 (1.73, 2.63) | <0.001 | 1.92 (1.52, 2.41) | <0.001 | 3.20 (2.05, 5.00) | <0.001 |
| G5 (<15) | 3.05 (2.15, 4.31) | <0.001 | 3.40 (2.40, 4.82) | <0.001 | 2.88 (1.19, 6.94) | 0.019 |
| ACR, mg/mmol | |  |  |  |  |  |
| A1 (<3) | 1.00 (reference) | reference | 1.00 (reference) | reference | 1.00 (reference) | reference |
| A2 (3-30) | 1.20 (1.15, 1.25) | <0.001 | 1.17 (1.12, 1.23) | <0.001 | 1.38 (1.25, 1.52) | <0.001 |
| A3 (>30) | 1.69 (1.52, 1.87) | <0.001 | 1.64 (1.46, 1.83) | <0.001 | 2.09 (1.68, 2.61) | <0.001 |
| eGFRcr-cys & ACR | |  |  |  |  |  |
| Low | 1.00 (reference) | reference | 1.00 (reference) | reference | 1.00 (reference) | reference |
| Moderate | 1.19 (1.14, 1.25) | <0.001 | 1.17 (1.12, 1.23) | <0.001 | 1.29 (1.17, 1.43) | <0.001 |
| High | 1.55 (1.41, 1.69) | <0.001 | 1.46 (1.32, 1.61) | <0.001 | 2.03 (1.67, 2.47) | <0.001 |
| Very high | 1.91 (1.69, 2.16) | <0.001 | 1.88 (1.65, 2.14) | <0.001 | 2.27 (1.74, 2.97) | <0.001 |

CHD, coronary heart disease; CI, confidence interval; CVD, cardiovascular disease; HR, hazard ratio, IS, ischemic stroke.

Multivariable model was adjusted for age, sex, income score, ethnic, Body mass index(BMI), qualification, smoking status, alcohol status, total physical activity level, duration of sleep, fruit consumption, processed meats consumption, vegetables consumption, fishes consumption, tea consumption, coffee consumption, family history of heart diseases or stroke (only in the corresponding analysis), prevalent hypertension, prevalent diabetes, high-density lipoprotein cholesterol (HDLc), low-density lipoprotein cholesterol (LDLc), use of antihypertensive drugs, use of antihyperlipidemic drugs and use of antidiabetic drugs, diuretic use.

## Table S15. Mediation effect [hazard ratio (95% CI)] for renal function in the association of urate with CVDs.

| Outcomes | Mediate | DE | IE | TE | Proportion Mediated (%) |
| --- | --- | --- | --- | --- | --- |
| CVD | eGFRcr-cys | **1.050 (1.036, 1.064)** | **1.038 (1.034, 1.043)** | **1.090 (1.077, 1.104)** | 43.563 (36.198, 52.815) |
| CHD | eGFRcr-cys | **1.051 (1.036, 1.066)** | **1.038 (1.033, 1.043)** | **1.091 (1.077, 1.106)** | 42.641 (34.849, 52.569) |
| IS | eGFRcr-cys | **1.053 (1.018, 1.088)** | **1.057 (1.045, 1.069)** | **1.113 (1.079, 1.147)** | 51.837 (36.624, 76.840) |
| CVD | ACR | **1.091 (1.071, 1.111)** | **1.002 (1.002, 1.003)** | **1.093 (1.074, 1.113)** | 2.485 (1.760, 3.435) |
| CHD | ACR | **1.096 (1.074, 1.117)** | **1.002 (1.002, 1.003)** | **1.098 (1.077, 1.120)** | 2.314 (1.576, 3.283) |
| IS | ACR | **1.115 (1.067, 1.164)** | **1.002 (1.001, 1.004)** | **1.117 (1.070, 1.167)** | 2.114 (0.970, 4.015) |

CHD, coronary heart disease; CI, confidence interval; CVD, cardiovascular disease; HR, hazard ratio, IS, ischemic stroke.

Multivariable model was adjusted for age, sex, income score, ethnic, Body mass index(BMI), qualification, smoking status, alcohol status, total physical activity level, duration of sleep, fruit consumption, processed meats consumption, vegetables consumption, fishes consumption, tea consumption, coffee consumption, family history of heart diseases or stroke (only in the corresponding analysis), prevalent hypertension, high-density lipoprotein cholesterol (HDLc), low-density lipoprotein cholesterol (LDLc), use of antihypertensive drugs, use of antihyperlipidemic drugs and use of antidiabetic drugs. Significant results are indicated in bold.

## Table S16. Additive interaction between urate and kidney damage markers on CVDs risk.

| Outcome | Urate | Renal function | RERI | AP |
| --- | --- | --- | --- | --- |
| eGFRcr-cys | |  |  |  |
| CVD | Q2 | eGFRcr-cys 60-89 | 0.01 (-0.08, 0.10) | 0.01 (-0.07, 0.08) |
| CVD | Q2 | eGFRcr-cys <60 | -0.04 (-0.67, 0.58) | -0.03 (-0.40, 0.34) |
| CVD | Q3 | eGFRcr-cys 60-89 | -0.02 (-0.11, 0.06) | -0.02 (-0.09, 0.05) |
| CVD | Q3 | eGFRcr-cys <60 | -0.01 (-0.58, 0.57) | -0.00 (-0.32, 0.31) |
| CHD | Q2 | eGFRcr-cys 60-89 | 0.01 (-0.09, 0.10) | 0.00 (-0.08, 0.09) |
| CHD | Q2 | eGFRcr-cys <60 | -0.19 (-0.90, 0.52) | -0.11 (-0.54, 0.32) |
| CHD | Q3 | eGFRcr-cys 60-89 | -0.04 (-0.13, 0.05) | -0.03 (-0.11, 0.04) |
| CHD | Q3 | eGFRcr-cys <60 | -0.16 (-0.81, 0.50) | -0.09 (-0.45, 0.27) |
| IS | Q2 | eGFRcr-cys 60-89 | 0.05 (-0.17, 0.28) | 0.04 (-0.13, 0.22) |
| IS | Q2 | eGFRcr-cys <60 | -0.23 (-2.03, 1.57) | -0.11 (-0.95, 0.73) |
| IS | Q3 | eGFRcr-cys 60-89 | 0.03 (-0.19, 0.24) | 0.02 (-0.14, 0.18) |
| IS | Q3 | eGFRcr-cys <60 | -0.47 (-2.13, 1.20) | -0.23 (-1.05, 0.59) |
| ACR |  |  |  |  |
| CVD | Q2 | ACR 3-30 | 0.04 (-0.15, 0.23) | 0.03 (-0.12, 0.18) |
| CVD | Q2 | ACR > 30 | -0.93 (-1.93, 0.07) | -0.58 (-1.31, 0.15) |
| CVD | Q3 | ACR 3-30 | 0.06 (-0.12, 0.24) | 0.04 (-0.08, 0.17) |
| CVD | Q3 | ACR > 30 | -0.56 (-1.48, 0.37) | -0.27 (-0.72, 0.19) |
| CHD | Q2 | ACR 3-30 | 0.05 (-0.16, 0.26) | 0.04 (-0.12, 0.20) |
| CHD | Q2 | ACR > 30 | -0.47 (-1.44, 0.50) | -0.30 (-1.00, 0.39) |
| CHD | Q3 | ACR 3-30 | 0.09 (-0.10, 0.29) | 0.07 (-0.07, 0.20) |
| CHD | Q3 | ACR > 30 | 0.01 (-0.86, 0.89) | 0.01 (-0.41, 0.42) |
| IS | Q2 | ACR 3-30 | 0.26 (-0.23, 0.76) | 0.16 (-0.12, 0.44) |
| IS | Q2 | ACR > 30 | -2.06 (-4.97, 0.86) | -1.01 (-2.83, 0.80) |
| IS | Q3 | ACR 3-30 | 0.07 (-0.39, 0.52) | 0.04 (-0.24, 0.32) |
| IS | Q3 | ACR > 30 | -1.82 (-4.54, 0.90) | -0.77 (-2.01, 0.47) |
| eGFRcr-cys & ACR | |  |  |  |
| CVD | Q2 | Moderate risk | 0.04 (-0.17, 0.24) | 0.03 (-0.13, 0.18) |
| CVD | Q2 | High risk | -0.77 (-1.67, 0.13) | -0.54 (-1.26, 0.18) |
| CVD | Q2 | Very high risk | -0.33 (-2.78, 2.12) | -0.12 (-1.04, 0.80) |
| CVD | Q3 | Moderate risk | 0.04 (-0.15, 0.23) | 0.03 (-0.11, 0.16) |
| CVD | Q3 | High risk | -0.42 (-1.25, 0.41) | -0.23 (-0.68, 0.23) |
| CVD | Q3 | Very high risk | -0.84 (-3.00, 1.31) | -0.36 (-1.30, 0.58) |
| CHD | Q2 | Moderate risk | 0.05 (-0.17, 0.27) | 0.04 (-0.13, 0.21) |
| CHD | Q2 | High risk | -0.51 (-1.39, 0.37) | -0.40 (-1.16, 0.36) |
| CHD | Q2 | Very high risk | -0.41 (-2.94, 2.12) | -0.16 (-1.15, 0.84) |
| CHD | Q3 | Moderate risk | 0.07 (-0.13, 0.28) | 0.05 (-0.09, 0.20) |
| CHD | Q3 | High risk | -0.05 (-0.85, 0.76) | -0.02 (-0.47, 0.42) |
| CHD | Q3 | Very high risk | -0.80 (-3.02, 1.41) | -0.34 (-1.30, 0.62) |
| IS | Q2 | Moderate risk | 0.19 (-0.32, 0.70) | 0.12 (-0.19, 0.43) |
| IS | Q2 | High risk | -1.33 (-4.09, 1.43) | -0.57 (-1.93, 0.79) |
| IS | Q2 | Very high risk | 1.09 (-4.36, 6.55) | 0.31 (-1.08, 1.69) |
| IS | Q3 | Moderate risk | -0.05 (-0.51, 0.42) | -0.03 (-0.36, 0.30) |
| IS | Q3 | High risk | -1.56 (-4.10, 0.99) | -0.71 (-1.95, 0.52) |
| IS | Q3 | Very high risk | 0.06 (-4.51, 4.63) | 0.02 (-1.71, 1.75) |

AP, attributable proportion due to interaction; CHD, coronary heart disease; CI, confidence interval; CVD, cardiovascular disease; HR, hazard ratio; IS, ischemic stroke; RERI, relative excess risk due to interaction.

Multivariable model was adjusted for age, sex, income score, ethnic, Body mass index(BMI), qualification, smoking status, alcohol status, total physical activity level, duration of sleep, fruit consumption, processed meats consumption, vegetables consumption, fishes consumption, tea consumption, coffee consumption, family history of heart diseases or stroke (only in the corresponding analysis), prevalent hypertension, prevalent diabetes, high-density lipoprotein cholesterol (HDLc), low-density lipoprotein cholesterol (LDLc), use of antihypertensive drugs, use of antihyperlipidemic drugs and use of antidiabetic drugs. The estimates of RERI and AP were calculated based on the reference group with the lowest tertile of urate and the lowest level of relative kidney damage biomarker.

## Table S17. Joint associations of urate and renal function with CVD outcomes after excluding baseline diabetic patients.

| Group | CVD | | CHD | | IS | |
| --- | --- | --- | --- | --- | --- | --- |
|  | HR (95% CI) | *P* | HR (95% CI) | *P* | HR (95% CI) | *P* |
| eGFRcr-cys >=90 |  |  |  |  |  |  |
| Q1 | 1.00 (reference) | reference | 1.00 (reference) | reference | 1.00 (reference) | reference |
| Q2 | 1.04 (1.00, 1.09) | 0.04 | 1.06 (1.01, 1.11) | 0.016 | 1.02 (0.92, 1.14) | 0.69 |
| Q3 | 1.15 (1.10, 1.20) | <0.001 | 1.17 (1.12, 1.23) | <0.001 | 1.13 (1.01, 1.27) | 0.035 |
| eGFRcr-cys 60-89 |  |  |  |  |  |  |
| Q1 | 1.11 (1.05, 1.17) | <0.001 | 1.11 (1.05, 1.18) | <0.001 | 1.19 (1.04, 1.36) | 0.012 |
| Q2 | 1.17 (1.12, 1.22) | <0.001 | 1.17 (1.12, 1.23) | <0.001 | 1.28 (1.15, 1.43) | <0.001 |
| Q3 | 1.24 (1.19, 1.29) | <0.001 | 1.24 (1.18, 1.30) | <0.001 | 1.34 (1.20, 1.50) | <0.001 |
| eGFRcr-cys <60 |  |  |  |  |  |  |
| Q1 | 1.56 (1.14, 2.12) | 0.005 | 1.61 (1.15, 2.25) | 0.006 | 2.19 (1.13, 4.23) | 0.02 |
| Q2 | 1.61 (1.37, 1.89) | <0.001 | 1.53 (1.28, 1.83) | <0.001 | 2.53 (1.83, 3.50) | <0.001 |
| Q3 | 1.75 (1.62, 1.90) | <0.001 | 1.71 (1.56, 1.86) | <0.001 | 2.00 (1.65, 2.44) | <0.001 |
| ACR < 3 |  |  |  |  |  |  |
| Q1 | 1.00 (reference) | reference | 1.00 (reference) | reference | 1.00 (reference) | reference |
| Q2 | 1.11 (1.04, 1.18) | 0.001 | 1.11 (1.04, 1.20) | 0.003 | 1.16 (0.99, 1.36) | 0.058 |
| Q3 | 1.21 (1.13, 1.29) | <0.001 | 1.22 (1.13, 1.31) | <0.001 | 1.24 (1.05, 1.45) | 0.009 |
| ACR 3-30 |  |  |  |  |  |  |
| Q1 | 1.20 (1.07, 1.35) | 0.003 | 1.19 (1.04, 1.36) | 0.013 | 1.19 (0.90, 1.58) | 0.213 |
| Q2 | 1.32 (1.19, 1.46) | <0.001 | 1.27 (1.13, 1.42) | <0.001 | 1.73 (1.39, 2.15) | <0.001 |
| Q3 | 1.38 (1.27, 1.51) | <0.001 | 1.37 (1.24, 1.50) | <0.001 | 1.54 (1.25, 1.89) | <0.001 |
| ACR > 30 |  |  |  |  |  |  |
| Q1 | 2.27 (1.52, 3.40) | <0.001 | 1.70 (1.02, 2.84) | 0.04 | 4.85 (2.49, 9.43) | <0.001 |
| Q2 | 1.46 (1.05, 2.02) | 0.024 | 1.31 (0.90, 1.93) | 0.161 | 2.03 (1.04, 3.95) | 0.038 |
| Q3 | 1.78 (1.49, 2.13) | <0.001 | 1.88 (1.56, 2.27) | <0.001 | 1.49 (0.93, 2.39) | 0.098 |
| CKD risk low | |  |  |  |  |  |
| Q1 | 1.00 (reference) | reference | 1.00 (reference) | reference | 1.00 (reference) | reference |
| Q2 | 1.11 (1.04, 1.18) | 0.002 | 1.12 (1.04, 1.20) | 0.003 | 1.15 (0.98, 1.35) | 0.078 |
| Q3 | 1.19 (1.12, 1.28) | <0.001 | 1.21 (1.12, 1.30) | <0.001 | 1.22 (1.04, 1.44) | 0.015 |
| CKD risk moderate | |  |  |  |  |  |
| Q1 | 1.22 (1.09, 1.38) | <0.001 | 1.22 (1.07, 1.39) | 0.004 | 1.20 (0.91, 1.58) | 0.206 |
| Q2 | 1.32 (1.20, 1.46) | <0.001 | 1.28 (1.15, 1.43) | <0.001 | 1.61 (1.29, 2.01) | <0.001 |
| Q3 | 1.37 (1.26, 1.49) | <0.001 | 1.37 (1.24, 1.50) | <0.001 | 1.44 (1.18, 1.77) | <0.001 |
| CKD risk high | |  |  |  |  |  |
| Q1 | 1.89 (1.28, 2.79) | 0.001 | 1.61 (1.01, 2.56) | 0.046 | 3.41 (1.68, 6.89) | <0.001 |
| Q2 | 1.37 (1.03, 1.84) | 0.032 | 1.09 (0.76, 1.56) | 0.625 | 2.87 (1.75, 4.70) | <0.001 |
| Q3 | 1.74 (1.50, 2.02) | <0.001 | 1.68 (1.43, 1.99) | <0.001 | 1.74 (1.20, 2.52) | 0.003 |
| CKD risk very high | |  |  |  |  |  |
| Q1 | 2.09 (0.94, 4.68) | 0.071 | 1.91 (0.79, 4.60) | 0.149 | 2.56 (0.36, 18.32) | 0.348 |
| Q2 | 2.23 (1.46, 3.41) | <0.001 | 2.07 (1.28, 3.35) | 0.003 | 3.44 (1.52, 7.76) | 0.003 |
| Q3 | 2.02 (1.67, 2.44) | <0.001 | 2.12 (1.74, 2.59) | <0.001 | 2.05 (1.29, 3.25) | 0.002 |

CHD, coronary heart disease; CI, confidence interval; CVD, cardiovascular disease; HR, hazard ratio, IS, ischemic stroke.

Multivariable model was adjusted for age, sex, income score, ethnic, Body mass index(BMI), qualification, smoking status, alcohol status, total physical activity level, duration of sleep, fruit consumption, processed meats consumption, vegetables consumption, fishes consumption, tea consumption, coffee consumption, family history of heart diseases or stroke (only in the corresponding analysis), prevalent hypertension, high-density lipoprotein cholesterol (HDLc), low-density lipoprotein cholesterol (LDLc), use of antihypertensive drugs, use of antihyperlipidemic drugs and use of antidiabetic drugs.

## Table S18. Joint associations of urate and renal function with CVD outcomes after additionally adjusted for environmental stressors and well-being in life.

| Group | CVD | | CHD | | IS | |
| --- | --- | --- | --- | --- | --- | --- |
|  | HR (95% CI) | *P* | HR (95% CI) | *P* | HR (95% CI) | *P* |
| eGFRcr-cys >=90 |  |  |  |  |  |  |
| Q1 | 1.00 (reference) | reference | 1.00 (reference) | reference | 1.00 (reference) | reference |
| Q2 | 1.04 (1.00, 1.08) | 0.058 | 1.05 (1.01, 1.10) | 0.015 | 1.00 (0.91, 1.11) | 0.949 |
| Q3 | 1.15 (1.10, 1.20) | <0.001 | 1.17 (1.12, 1.23) | <0.001 | 1.10 (0.99, 1.22) | 0.084 |
| eGFRcr-cys 60-89 |  |  |  |  |  |  |
| Q1 | 1.12 (1.06, 1.18) | <0.001 | 1.12 (1.06, 1.18) | <0.001 | 1.21 (1.07, 1.37) | 0.002 |
| Q2 | 1.16 (1.12, 1.21) | <0.001 | 1.18 (1.12, 1.23) | <0.001 | 1.27 (1.15, 1.40) | <0.001 |
| Q3 | 1.24 (1.20, 1.29) | <0.001 | 1.25 (1.20, 1.30) | <0.001 | 1.33 (1.21, 1.47) | <0.001 |
| eGFRcr-cys <60 |  |  |  |  |  |  |
| Q1 | 1.72 (1.33, 2.22) | <0.001 | 1.84 (1.39, 2.42) | <0.001 | 2.42 (1.42, 4.11) | 0.001 |
| Q2 | 1.70 (1.48, 1.94) | <0.001 | 1.68 (1.45, 1.95) | <0.001 | 2.18 (1.63, 2.92) | <0.001 |
| Q3 | 1.85 (1.73, 1.97) | <0.001 | 1.83 (1.70, 1.97) | <0.001 | 2.04 (1.74, 2.40) | <0.001 |
| ACR < 3 |  |  |  |  |  |  |
| Q1 | 1.00 (reference) | reference | 1.00 (reference) | reference | 1.00 (reference) | reference |
| Q2 | 1.09 (1.03, 1.15) | 0.004 | 1.09 (1.03, 1.17) | 0.006 | 1.11 (0.96, 1.28) | 0.152 |
| Q3 | 1.20 (1.13, 1.27) | <0.001 | 1.21 (1.13, 1.29) | <0.001 | 1.21 (1.05, 1.40) | 0.007 |
| ACR 3-30 |  |  |  |  |  |  |
| Q1 | 1.19 (1.07, 1.31) | 0.001 | 1.14 (1.01, 1.28) | 0.029 | 1.32 (1.05, 1.66) | 0.017 |
| Q2 | 1.31 (1.21, 1.43) | <0.001 | 1.28 (1.17, 1.41) | <0.001 | 1.70 (1.41, 2.05) | <0.001 |
| Q3 | 1.44 (1.34, 1.56) | <0.001 | 1.44 (1.33, 1.56) | <0.001 | 1.60 (1.35, 1.91) | <0.001 |
| ACR > 30 |  |  |  |  |  |  |
| Q1 | 2.47 (1.87, 3.27) | <0.001 | 1.94 (1.39, 2.71) | <0.001 | 4.01 (2.38, 6.73) | <0.001 |
| Q2 | 1.60 (1.25, 2.05) | <0.001 | 1.54 (1.17, 2.03) | 0.002 | 2.04 (1.22, 3.44) | 0.007 |
| Q3 | 2.09 (1.83, 2.37) | <0.001 | 2.13 (1.86, 2.45) | <0.001 | 2.36 (1.76, 3.16) | <0.001 |
| CKD risk low | |  |  |  |  |  |
| Q1 | 1.00 (reference) | reference | 1.00 (reference) | reference | 1.00 (reference) | reference |
| Q2 | 1.09 (1.02, 1.15) | 0.006 | 1.09 (1.03, 1.17) | 0.006 | 1.10 (0.96, 1.27) | 0.186 |
| Q3 | 1.18 (1.11, 1.25) | <0.001 | 1.19 (1.12, 1.27) | <0.001 | 1.19 (1.04, 1.38) | 0.014 |
| CKD risk moderate | |  |  |  |  |  |
| Q1 | 1.19 (1.08, 1.32) | <0.001 | 1.15 (1.02, 1.29) | 0.018 | 1.29 (1.03, 1.63) | 0.03 |
| Q2 | 1.31 (1.21, 1.43) | <0.001 | 1.29 (1.18, 1.42) | <0.001 | 1.58 (1.31, 1.91) | <0.001 |
| Q3 | 1.41 (1.31, 1.52) | <0.001 | 1.41 (1.30, 1.53) | <0.001 | 1.44 (1.21, 1.72) | <0.001 |
| CKD risk high | |  |  |  |  |  |
| Q1 | 2.13 (1.61, 2.82) | <0.001 | 1.72 (1.23, 2.41) | 0.002 | 3.57 (2.12, 5.99) | <0.001 |
| Q2 | 1.41 (1.13, 1.77) | 0.003 | 1.28 (0.99, 1.65) | 0.061 | 2.32 (1.51, 3.55) | <0.001 |
| Q3 | 1.86 (1.66, 2.09) | <0.001 | 1.84 (1.63, 2.09) | <0.001 | 2.18 (1.68, 2.84) | <0.001 |
| CKD risk very high | |  |  |  |  |  |
| Q1 | 3.08 (1.82, 5.21) | <0.001 | 3.05 (1.76, 5.27) | <0.001 | 2.46 (0.61, 9.89) | 0.206 |
| Q2 | 2.75 (1.97, 3.85) | <0.001 | 2.63 (1.82, 3.81) | <0.001 | 3.58 (1.84, 6.97) | <0.001 |
| Q3 | 2.32 (2.02, 2.67) | <0.001 | 2.34 (2.01, 2.71) | <0.001 | 2.60 (1.90, 3.55) | <0.001 |

CHD, coronary heart disease; CI, confidence interval; CVD, cardiovascular disease; HR, hazard ratio, IS, ischemic stroke.

Multivariable model was adjusted for age, sex, income score, ethnic, Body mass index(BMI), qualification, smoking status, alcohol status, total physical activity level, duration of sleep, fruit consumption, processed meats consumption, vegetables consumption, fishes consumption, tea consumption, coffee consumption, family history of heart diseases or stroke (only in the corresponding analysis), prevalent hypertension, prevalent diabetes, high-density lipoprotein cholesterol (HDLc), low-density lipoprotein cholesterol (LDLc), use of antihypertensive drugs, use of antihyperlipidemic drugs and use of antidiabetic drugs, residential noise pollution, PM10, and well-being in life.

## Table S19. Joint associations of urate and renal function with CVD outcomes after excluding participants with incident CVDs in the previous 2 years.

| Group | CVD | | CHD | | IS | |
| --- | --- | --- | --- | --- | --- | --- |
|  | HR (95% CI) | *P* | HR (95% CI) | *P* | HR (95% CI) | *P* |
| eGFRcr-cys >=90 |  |  |  |  |  |  |
| Q1 | 1.00 (reference) | reference | 1.00 (reference) | reference | 1.00 (reference) | reference |
| Q2 | 1.04 (1.00, 1.08) | 0.047 | 1.05 (1.01, 1.10) | 0.023 | 1.02 (0.92, 1.13) | 0.688 |
| Q3 | 1.14 (1.09, 1.19) | <0.001 | 1.16 (1.10, 1.21) | <0.001 | 1.10 (0.99, 1.23) | 0.076 |
| eGFRcr-cys 60-89 |  |  |  |  |  |  |
| Q1 | 1.12 (1.06, 1.18) | <0.001 | 1.12 (1.05, 1.19) | <0.001 | 1.18 (1.04, 1.35) | 0.011 |
| Q2 | 1.16 (1.11, 1.21) | <0.001 | 1.17 (1.11, 1.23) | <0.001 | 1.25 (1.13, 1.40) | <0.001 |
| Q3 | 1.23 (1.18, 1.28) | <0.001 | 1.23 (1.17, 1.29) | <0.001 | 1.31 (1.19, 1.46) | <0.001 |
| eGFRcr-cys <60 |  |  |  |  |  |  |
| Q1 | 1.64 (1.24, 2.18) | <0.001 | 1.70 (1.25, 2.31) | <0.001 | 2.45 (1.42, 4.25) | 0.001 |
| Q2 | 1.73 (1.50, 1.99) | <0.001 | 1.70 (1.46, 1.99) | <0.001 | 2.15 (1.58, 2.92) | <0.001 |
| Q3 | 1.83 (1.70, 1.97) | <0.001 | 1.80 (1.66, 1.94) | <0.001 | 2.07 (1.75, 2.45) | <0.001 |
| ACR < 3 |  |  |  |  |  |  |
| Q1 | 1.00 (reference) | reference | 1.00 (reference) | reference | 1.00 (reference) | reference |
| Q2 | 1.09 (1.03, 1.16) | 0.004 | 1.08 (1.01, 1.16) | 0.023 | 1.16 (1.00, 1.35) | 0.044 |
| Q3 | 1.19 (1.12, 1.27) | <0.001 | 1.19 (1.11, 1.27) | <0.001 | 1.25 (1.08, 1.46) | 0.003 |
| ACR 3-30 |  |  |  |  |  |  |
| Q1 | 1.20 (1.08, 1.33) | 0.001 | 1.13 (1.00, 1.28) | 0.051 | 1.40 (1.10, 1.77) | 0.006 |
| Q2 | 1.31 (1.20, 1.43) | <0.001 | 1.27 (1.15, 1.40) | <0.001 | 1.71 (1.40, 2.08) | <0.001 |
| Q3 | 1.40 (1.29, 1.51) | <0.001 | 1.38 (1.26, 1.50) | <0.001 | 1.60 (1.33, 1.92) | <0.001 |
| ACR > 30 |  |  |  |  |  |  |
| Q1 | 2.39 (1.76, 3.25) | <0.001 | 1.82 (1.26, 2.63) | 0.001 | 4.23 (2.47, 7.25) | <0.001 |
| Q2 | 1.42 (1.07, 1.88) | 0.016 | 1.32 (0.96, 1.81) | 0.090 | 1.83 (1.03, 3.27) | 0.040 |
| Q3 | 2.04 (1.77, 2.35) | <0.001 | 2.06 (1.77, 2.40) | <0.001 | 2.52 (1.87, 3.41) | <0.001 |
| CKD risk low | |  |  |  |  |  |
| Q1 | 1.00 (reference) | reference | 1.00 (reference) | reference | 1.00 (reference) | reference |
| Q2 | 1.09 (1.03, 1.16) | 0.005 | 1.08 (1.01, 1.16) | 0.025 | 1.16 (1.00, 1.34) | 0.055 |
| Q3 | 1.18 (1.10, 1.25) | <0.001 | 1.17 (1.10, 1.26) | <0.001 | 1.24 (1.06, 1.44) | 0.005 |
| CKD risk moderate | |  |  |  |  |  |
| Q1 | 1.20 (1.08, 1.34) | <0.001 | 1.14 (1.01, 1.29) | 0.036 | 1.37 (1.07, 1.74) | 0.011 |
| Q2 | 1.31 (1.19, 1.43) | <0.001 | 1.27 (1.15, 1.41) | <0.001 | 1.61 (1.31, 1.97) | <0.001 |
| Q3 | 1.35 (1.25, 1.46) | <0.001 | 1.34 (1.23, 1.46) | <0.001 | 1.42 (1.18, 1.72) | <0.001 |
| CKD risk high | |  |  |  |  |  |
| Q1 | 2.19 (1.63, 2.95) | <0.001 | 1.78 (1.25, 2.53) | 0.001 | 3.77 (2.20, 6.46) | <0.001 |
| Q2 | 1.31 (1.02, 1.68) | 0.037 | 1.18 (0.89, 1.57) | 0.258 | 2.05 (1.27, 3.31) | 0.003 |
| Q3 | 1.88 (1.66, 2.13) | <0.001 | 1.84 (1.61, 2.11) | <0.001 | 2.36 (1.80, 3.09) | <0.001 |
| CKD risk very high | |  |  |  |  |  |
| Q1 | 2.59 (1.39, 4.82) | 0.003 | 2.44 (1.26, 4.71) | 0.008 | 2.72 (0.67, 10.95) | 0.160 |
| Q2 | 2.80 (1.95, 4.02) | <0.001 | 2.55 (1.70, 3.83) | <0.001 | 3.65 (1.80, 7.39) | <0.001 |
| Q3 | 2.30 (1.98, 2.68) | <0.001 | 2.26 (1.92, 2.67) | <0.001 | 2.74 (1.98, 3.79) | <0.001 |

CHD, coronary heart disease; CI, confidence interval; CVD, cardiovascular disease; HR, hazard ratio, IS, ischemic stroke.

Multivariable model was adjusted for age, sex, income score, ethnic, Body mass index(BMI), qualification, smoking status, alcohol status, total physical activity level, duration of sleep, fruit consumption, processed meats consumption, vegetables consumption, fishes consumption, tea consumption, coffee consumption, family history of heart diseases or stroke (only in the corresponding analysis), prevalent hypertension, high-density lipoprotein cholesterol (HDLc), low-density lipoprotein cholesterol (LDLc), use of antihypertensive drugs, use of antihyperlipidemic drugs and use of antidiabetic drugs.

## Table S20. Joint associations of urate and renal function with CVD outcomes stratified by ethnicity.

| Ethnic | Group | CVD | | CHD | | IS | |
| --- | --- | --- | --- | --- | --- | --- | --- |
|  |  | HR (95% CI) | *P* | HR (95% CI) | *P* | HR (95% CI) | *P* |
| White | eGFRcr-cys >=90 |  |  |  |  |  |  |
|  | Q1 | 1.00 (reference) | reference | 1.00 (reference) | reference | 1.00 (reference) | reference |
|  | Q2 | 1.05 (1.00, 1.09) | 0.031 | 1.07 (1.02, 1.12) | 0.004 | 0.97 (0.88, 1.08) | 0.618 |
|  | Q3 | 1.15 (1.11, 1.20) | <0.001 | 1.18 (1.13, 1.24) | <0.001 | 1.09 (0.97, 1.21) | 0.147 |
|  | eGFRcr-cys 60-89 |  |  |  |  |  |  |
|  | Q1 | 1.12 (1.06, 1.18) | <0.001 | 1.12 (1.05, 1.19) | <0.001 | 1.17 (1.03, 1.34) | 0.014 |
|  | Q2 | 1.17 (1.12, 1.22) | <0.001 | 1.19 (1.13, 1.25) | <0.001 | 1.29 (1.16, 1.43) | <0.001 |
|  | Q3 | 1.25 (1.20, 1.30) | <0.001 | 1.26 (1.20, 1.32) | <0.001 | 1.33 (1.20, 1.48) | <0.001 |
|  | eGFRcr-cys <60 |  |  |  |  |  |  |
|  | Q1 | 1.78 (1.37, 2.32) | <0.001 | 1.89 (1.43, 2.51) | <0.001 | 2.60 (1.53, 4.42) | <0.001 |
|  | Q2 | 1.71 (1.49, 1.97) | <0.001 | 1.68 (1.44, 1.96) | <0.001 | 2.19 (1.62, 2.96) | <0.001 |
|  | Q3 | 1.85 (1.72, 1.98) | <0.001 | 1.83 (1.70, 1.98) | <0.001 | 2.08 (1.76, 2.46) | <0.001 |
| Other | eGFRcr-cys >=90 |  |  |  |  |  |  |
|  | Q1 | 1.00 (reference) | reference | 1.00 (reference) | reference | 1.00 (reference) | reference |
|  | Q2 | 0.96 (0.84, 1.08) | 0.471 | 0.92 (0.80, 1.06) | 0.247 | 1.29 (0.93, 1.79) | 0.121 |
|  | Q3 | 1.08 (0.95, 1.23) | 0.239 | 1.07 (0.93, 1.24) | 0.341 | 1.19 (0.83, 1.70) | 0.337 |
|  | eGFRcr-cys 60-89 |  |  |  |  |  |  |
|  | Q1 | 1.16 (0.97, 1.39) | 0.107 | 1.11 (0.91, 1.35) | 0.321 | 1.75 (1.15, 2.66) | 0.009 |
|  | Q2 | 1.06 (0.92, 1.23) | 0.397 | 1.05 (0.90, 1.23) | 0.513 | 1.03 (0.70, 1.52) | 0.880 |
|  | Q3 | 1.19 (1.04, 1.36) | 0.009 | 1.16 (1.01, 1.35) | 0.038 | 1.31 (0.92, 1.86) | 0.130 |
|  | eGFRcr-cys <60 |  |  |  |  |  |  |
|  | Q1 | 0.84 (0.21, 3.39) | 0.81 | 1.03 (0.26, 4.15) | 0.964 | 0.00 (0.00, Inf) | 0.989 |
|  | Q2 | 1.53 (0.92, 2.52) | 0.099 | 1.73 (1.03, 2.91) | 0.04 | 2.16 (0.67, 6.94) | 0.197 |
|  | Q3 | 1.89 (1.50, 2.38) | <0.001 | 1.82 (1.42, 2.32) | <0.001 | 1.65 (0.88, 3.08) | 0.119 |
| White | ACR < 3 |  |  |  |  |  |  |
|  | Q1 | 1.00 (reference) | reference | 1.00 (reference) | reference | 1.00 (reference) | reference |
|  | Q2 | 1.11 (1.04, 1.18) | <0.001 | 1.12 (1.05, 1.20) | 0.001 | 1.09 (0.94, 1.26) | 0.248 |
|  | Q3 | 1.21 (1.14, 1.28) | <0.001 | 1.22 (1.14, 1.31) | <0.001 | 1.18 (1.02, 1.37) | 0.028 |
|  | ACR 3-30 |  |  |  |  |  |  |
|  | Q1 | 1.17 (1.05, 1.30) | 0.004 | 1.11 (0.99, 1.26) | 0.085 | 1.29 (1.01, 1.64) | 0.040 |
|  | Q2 | 1.32 (1.21, 1.44) | <0.001 | 1.30 (1.18, 1.44) | <0.001 | 1.63 (1.34, 1.99) | <0.001 |
|  | Q3 | 1.46 (1.35, 1.58) | <0.001 | 1.46 (1.34, 1.59) | <0.001 | 1.57 (1.31, 1.89) | <0.001 |
|  | ACR > 30 |  |  |  |  |  |  |
|  | Q1 | 2.74 (2.04, 3.67) | <0.001 | 2.18 (1.54, 3.08) | <0.001 | 3.91 (2.24, 6.82) | <0.001 |
|  | Q2 | 1.58 (1.21, 2.07) | <0.001 | 1.53 (1.14, 2.06) | 0.005 | 1.82 (1.02, 3.25) | 0.043 |
|  | Q3 | 2.07 (1.80, 2.38) | <0.001 | 2.12 (1.83, 2.46) | <0.001 | 2.34 (1.71, 3.20) | <0.001 |
| Other | ACR < 3 |  |  |  |  |  |  |
|  | Q1 | 1.00 (reference) | reference | 1.00 (reference) | reference | 1.00 (reference) | reference |
|  | Q2 | 0.91 (0.76, 1.10) | 0.344 | 0.89 (0.73, 1.10) | 0.29 | 1.33 (0.80, 2.22) | 0.274 |
|  | Q3 | 1.06 (0.88, 1.28) | 0.508 | 1.03 (0.84, 1.26) | 0.748 | 1.52 (0.92, 2.51) | 0.103 |
|  | ACR 3-30 |  |  |  |  |  |  |
|  | Q1 | 1.29 (0.95, 1.75) | 0.104 | 1.29 (0.92, 1.79) | 0.135 | 1.80 (0.83, 3.91) | 0.140 |
|  | Q2 | 1.20 (0.93, 1.55) | 0.166 | 1.09 (0.82, 1.45) | 0.537 | 2.31 (1.25, 4.29) | 0.008 |
|  | Q3 | 1.25 (1.00, 1.57) | 0.053 | 1.20 (0.94, 1.54) | 0.144 | 1.95 (1.08, 3.52) | 0.028 |
|  | ACR > 30 |  |  |  |  |  |  |
|  | Q1 | 1.06 (0.39, 2.88) | 0.902 | 0.62 (0.15, 2.51) | 0.502 | 5.86 (1.36, 25.23) | 0.018 |
|  | Q2 | 1.68 (0.86, 3.30) | 0.13 | 1.52 (0.71, 3.26) | 0.281 | 4.11 (1.20, 14.09) | 0.024 |
|  | Q3 | 2.08 (1.48, 2.92) | <0.001 | 2.08 (1.45, 2.99) | <0.001 | 2.71 (1.17, 6.26) | 0.020 |
| White | CKD risk low |  |  |  |  |  |  |
|  | Q1 | 1.00 (reference) | reference | 1.00 (reference) | reference | 1.00 (reference) | reference |
|  | Q2 | 1.11 (1.04, 1.17) | 0.001 | 1.12 (1.05, 1.20) | 0.001 | 1.08 (0.93, 1.25) | 0.301 |
|  | Q3 | 1.19 (1.12, 1.27) | <0.001 | 1.21 (1.13, 1.29) | <0.001 | 1.16 (1.00, 1.35) | 0.045 |
|  | CKD risk moderate |  |  |  |  |  |  |
|  | Q1 | 1.17 (1.05, 1.30) | 0.004 | 1.12 (0.99, 1.27) | 0.065 | 1.26 (0.98, 1.60) | 0.066 |
|  | Q2 | 1.32 (1.21, 1.44) | <0.001 | 1.31 (1.19, 1.44) | <0.001 | 1.52 (1.24, 1.86) | <0.001 |
|  | Q3 | 1.43 (1.32, 1.54) | <0.001 | 1.44 (1.32, 1.57) | <0.001 | 1.39 (1.16, 1.68) | <0.001 |
|  | CKD risk high |  |  |  |  |  |  |
|  | Q1 | 2.33 (1.74, 3.13) | <0.001 | 1.92 (1.36, 2.72) | <0.001 | 3.49 (2.00, 6.09) | <0.001 |
|  | Q2 | 1.40 (1.10, 1.79) | 0.006 | 1.29 (0.98, 1.70) | 0.067 | 2.24 (1.42, 3.53) | <0.001 |
|  | Q3 | 1.86 (1.64, 2.10) | <0.001 | 1.83 (1.60, 2.09) | <0.001 | 2.20 (1.67, 2.89) | <0.001 |
|  | CKD risk very high |  |  |  |  |  |  |
|  | Q1 | 3.19 (1.88, 5.41) | <0.001 | 3.17 (1.83, 5.49) | <0.001 | 2.49 (0.62, 10.04) | 0.199 |
|  | Q2 | 2.67 (1.88, 3.79) | <0.001 | 2.52 (1.70, 3.71) | <0.001 | 3.28 (1.62, 6.64) | <0.001 |
|  | Q3 | 2.35 (2.03, 2.73) | <0.001 | 2.41 (2.05, 2.83) | <0.001 | 2.55 (1.83, 3.57) | <0.001 |
| Other | CKD risk low |  |  |  |  |  |  |
|  | Q1 | 1.00 (reference) | reference | 1.00 (reference) | reference | 1.00 (reference) | reference |
|  | Q2 | 0.91 (0.75, 1.10) | 0.324 | 0.89 (0.72, 1.09) | 0.268 | 1.33 (0.80, 2.22) | 0.271 |
|  | Q3 | 1.05 (0.87, 1.26) | 0.623 | 1.03 (0.84, 1.26) | 0.807 | 1.46 (0.88, 2.43) | 0.142 |
|  | CKD risk moderate |  |  |  |  |  |  |
|  | Q1 | 1.32 (0.98, 1.79) | 0.07 | 1.33 (0.96, 1.85) | 0.09 | 1.79 (0.82, 3.89) | 0.142 |
|  | Q2 | 1.20 (0.93, 1.55) | 0.166 | 1.11 (0.84, 1.48) | 0.465 | 2.20 (1.18, 4.12) | 0.014 |
|  | Q3 | 1.23 (0.98, 1.55) | 0.076 | 1.16 (0.90, 1.49) | 0.25 | 1.95 (1.09, 3.51) | 0.025 |
|  | CKD risk high |  |  |  |  |  |  |
|  | Q1 | 0.99 (0.37, 2.68) | 0.986 | 0.58 (0.14, 2.36) | 0.45 | 5.42 (1.26, 23.34) | 0.023 |
|  | Q2 | 1.51 (0.80, 2.87) | 0.208 | 1.21 (0.56, 2.59) | 0.629 | 3.47 (1.02, 11.83) | 0.047 |
|  | Q3 | 1.87 (1.32, 2.65) | <0.001 | 1.92 (1.33, 2.77) | <0.001 | 2.12 (0.84, 5.35) | 0.112 |
|  | CKD risk very high |  |  |  |  |  |  |
|  | Q1 | 0.00 (0.00, Inf) | 0.983 | 0.00 (0.00, Inf) | 0.986 | 0.00 (0.00, Inf) | 0.993 |
|  | Q2 | 3.67 (1.16, 11.62) | 0.027 | 4.61 (1.45, 14.66) | 0.01 | 6.64 (0.85, 51.66) | 0.071 |
|  | Q3 | 2.09 (1.41, 3.10) | <0.001 | 1.88 (1.23, 2.87) | 0.004 | 3.69 (1.52, 8.97) | 0.004 |

CHD, coronary heart disease; CI, confidence interval; CVD, cardiovascular disease; HR, hazard ratio, IS, ischemic stroke.

Multivariable model was adjusted for age, sex, income score, Body mass index(BMI), qualification, smoking status, alcohol status, total physical activity level, duration of sleep, fruit consumption, processed meats consumption, vegetables consumption, fishes consumption, tea consumption, coffee consumption, family history of heart diseases or stroke (only in the corresponding analysis), prevalent hypertension, high-density lipoprotein cholesterol (HDLc), low-density lipoprotein cholesterol (LDLc), use of antihypertensive drugs, use of antihyperlipidemic drugs and use of antidiabetic drugs.

**Table S21. Joint associations of urate and renal function with CVD outcomes additionally adjusting for diuretic use.**

| Group | CVD | | CHD | | IS | |
| --- | --- | --- | --- | --- | --- | --- |
|  | HR (95% CI) | *P* | HR (95% CI) | *P* | HR (95% CI) | *P* |
| eGFRcr-cys >=90 | | | | | | |
| Q1 | 1.00 (reference) | reference | 1.00 (reference) | reference | 1.00 (reference) | reference |
| Q2 | 1.04 (1.00, 1.08) | 0.066 | 1.05 (1.01, 1.10) | 0.017 | 1.00 (0.91, 1.11) | 0.973 |
| Q3 | 1.14 (1.10, 1.19) | <0.001 | 1.17 (1.12, 1.22) | <0.001 | 1.09 (0.98, 1.21) | 0.1 |
| eGFRcr-cys 60-89 | | | | | | |
| Q1 | 1.12 (1.06, 1.17) | <0.001 | 1.12 (1.05, 1.18) | <0.001 | 1.21 (1.07, 1.37) | 0.002 |
| Q2 | 1.16 (1.11, 1.21) | <0.001 | 1.17 (1.12, 1.22) | <0.001 | 1.27 (1.14, 1.40) | <0.001 |
| Q3 | 1.23 (1.18, 1.28) | <0.001 | 1.23 (1.18, 1.29) | <0.001 | 1.32 (1.20, 1.46) | <0.001 |
| eGFRcr-cys <60 | | | | | | |
| Q1 | 1.61 (1.25, 2.09) | <0.001 | 1.71 (1.30, 2.26) | <0.001 | 2.33 (1.37, 3.96) | 0.002 |
| Q2 | 1.66 (1.45, 1.89) | <0.001 | 1.63 (1.41, 1.89) | <0.001 | 2.15 (1.61, 2.88) | <0.001 |
| Q3 | 1.69 (1.58, 1.81) | <0.001 | 1.66 (1.54, 1.79) | <0.001 | 1.93 (1.64, 2.28) | <0.001 |
| ACR < 3 |  |  |  |  |  |  |
| Q1 | 1.00 (reference) | reference | 1.00 (reference) | reference | 1.00 (reference) | reference |
| Q2 | 1.09 (1.02, 1.15) | 0.005 | 1.09 (1.02, 1.16) | 0.007 | 1.10 (0.96, 1.27) | 0.166 |
| Q3 | 1.18 (1.12, 1.26) | <0.001 | 1.19 (1.12, 1.27) | <0.001 | 1.20 (1.04, 1.38) | 0.011 |
| ACR 3-30 |  |  |  |  |  |  |
| Q1 | 1.18 (1.07, 1.31) | 0.001 | 1.14 (1.01, 1.27) | 0.03 | 1.32 (1.05, 1.66) | 0.019 |
| Q2 | 1.31 (1.21, 1.43) | <0.001 | 1.28 (1.17, 1.41) | <0.001 | 1.69 (1.40, 2.04) | <0.001 |
| Q3 | 1.41 (1.31, 1.52) | <0.001 | 1.40 (1.29, 1.52) | <0.001 | 1.57 (1.32, 1.87) | <0.001 |
| ACR > 30 |  |  |  |  |  |  |
| Q1 | 2.43 (1.83, 3.21) | <0.001 | 1.90 (1.36, 2.66) | <0.001 | 3.92 (2.33, 6.59) | <0.001 |
| Q2 | 1.56 (1.22, 2.00) | <0.001 | 1.50 (1.13, 1.98) | 0.004 | 2.01 (1.19, 3.38) | 0.009 |
| Q3 | 1.92 (1.68, 2.18) | <0.001 | 1.95 (1.70, 2.24) | <0.001 | 2.24 (1.67, 3.01) | <0.001 |
| CKD risk low | |  |  |  |  |  |
| Q1 | 1.00 (reference) | reference | 1.00 (reference) | reference | 1.00 (reference) | reference |
| Q2 | 1.08 (1.02, 1.15) | 0.007 | 1.09 (1.02, 1.17) | 0.007 | 1.10 (0.95, 1.26) | 0.2 |
| Q3 | 1.17 (1.10, 1.24) | <0.001 | 1.18 (1.11, 1.26) | <0.001 | 1.19 (1.03, 1.37) | 0.019 |
| CKD risk moderate | |  |  |  |  |  |
| Q1 | 1.19 (1.08, 1.32) | <0.001 | 1.15 (1.02, 1.29) | 0.017 | 1.29 (1.02, 1.62) | 0.032 |
| Q2 | 1.31 (1.20, 1.42) | <0.001 | 1.29 (1.17, 1.41) | <0.001 | 1.57 (1.30, 1.90) | <0.001 |
| Q3 | 1.38 (1.28, 1.49) | <0.001 | 1.38 (1.28, 1.50) | <0.001 | 1.42 (1.19, 1.69) | <0.001 |
| CKD risk high | |  |  |  |  |  |
| Q1 | 2.06 (1.56, 2.73) | <0.001 | 1.67 (1.19, 2.33) | 0.003 | 3.47 (2.07, 5.84) | <0.001 |
| Q2 | 1.40 (1.12, 1.76) | 0.003 | 1.27 (0.98, 1.64) | 0.07 | 2.31 (1.51, 3.54) | <0.001 |
| Q3 | 1.77 (1.58, 1.99) | <0.001 | 1.74 (1.54, 1.97) | <0.001 | 2.12 (1.63, 2.75) | <0.001 |
| CKD risk very high | |  |  |  |  |  |
| Q1 | 2.66 (1.57, 4.51) | <0.001 | 2.63 (1.52, 4.56) | <0.001 | 2.22 (0.55, 8.95) | 0.262 |
| Q2 | 2.70 (1.93, 3.77) | <0.001 | 2.60 (1.80, 3.76) | <0.001 | 3.54 (1.82, 6.89) | <0.001 |
| Q3 | 2.05 (1.78, 2.36) | <0.001 | 2.04 (1.76, 2.38) | <0.001 | 2.43 (1.77, 3.33) | <0.001 |

CHD, coronary heart disease; CI, confidence interval; CVD, cardiovascular disease; HR, hazard ratio, IS, ischemic stroke.

Multivariable model was adjusted for age, sex, income score, ethnic, Body mass index(BMI), qualification, smoking status, alcohol status, total physical activity level, duration of sleep, fruit consumption, processed meats consumption, vegetables consumption, fishes consumption, tea consumption, coffee consumption, family history of heart diseases or stroke (only in the corresponding analysis), prevalent hypertension, high-density lipoprotein cholesterol (HDLc), low-density lipoprotein cholesterol (LDLc), use of antihypertensive drugs, use of antihyperlipidemic drugs and use of antidiabetic drugs, diuretic use.

## Table S22. Multivariable-adjusted HRs (95%CIs) for incident cardiovascular diseases by genetic risk among 343,054 European ancestry participants.

| **Genetic risk** | | | **HR (95%CI)** | | ***P*** | | ***P* for trend** |
| --- | --- | --- | --- | --- | --- | --- | --- |
| **CHD** | | |  | |  | |  |
| Low genetic risk | | | 1.00 (reference) | | reference | | <0.001 |
| Intermediate genetic risk | | | **1.22(1.18,1.26)** | | <0.001 | |  |
| High genetic risk | | | **1.57(1.51,1.63)** | | <0.001 | |  |
| **Ischemic Stroke** |  |  | |  | |  |  |
| Low genetic risk | | | 1.00 (reference) | | reference | | <0.001 |
| Intermediate genetic risk | | | **1.14(1.06,1.23)** | | <0.001 | |  |
| High genetic risk | | | **1.27(1.17,1.39)** | | <0.001 | |  |

CHD, coronary heart disease; CI, confidence interval; HR, hazard ratio.

Multivariable model was adjusted for age, sex, income score, Body mass index(BMI), qualification, smoking status, alcohol status, total physical activity level, duration of sleep, fruit consumption, processed meats consumption, vegetables consumption, fishes consumption, tea consumption, coffee consumption, family history of heart diseases or stroke (only in the corresponding analysis), prevalent hypertension, prevalent diabetes, high-density lipoprotein cholesterol (HDLc), low-density lipoprotein cholesterol (LDLc), use of antihypertensive drugs, use of antihyperlipidemic drugs and use of antidiabetic drugs. Significant results are indicated in bold (*P* < 0.05).

## Table S23. Primary analysis and sensitivity analyses of multiplicative interaction between genetic risk and serum urate with CHD.

| Genetic risk | Serum urate | Primary analysis | | Sensitivity analysis 1 | | Sensitivity analysis 2 | | Sensitivity analysis 3 | | Sensitivity analysis 4 | | Sensitivity analysis 5 | |
| --- | --- | --- | --- | --- | --- | --- | --- | --- | --- | --- | --- | --- | --- |
|  |  | HR (95%CI) | *P* for interaction | HR (95%CI) | *P* for interaction | HR (95%CI) | *P* for interaction | HR (95%CI) | *P* for interaction | HR (95%CI) | *P* for interaction | HR (95%CI) | *P* for interaction |
| Low |  |  | 0.020 |  | 0.016 |  | 0.020 |  | 0.047 |  | 0.043 |  | 0.041 |
|  | Q1 | Reference |  | Reference | | Reference | | Reference | | Reference | | Reference | |
|  | Q2 | 1.00(0.92,1.10) | | 1.04 (0.95, 1.15) | | 1.00 (0.92, 1.10) | | 1.02 (0.93, 1.12) | | 1.03 (0.95, 1.13) | | 1.03 (0.95, 1.13) | |
|  | Q3 | **1.13(1.03,1.23)** | | **1.17 (1.06, 1.30)** | | **1.13 (1.03, 1.23)** | | **1.12 (1.01, 1.24)** | | **1.13 (1.03, 1.24)** | | **1.14 (1.04, 1.25)** | |
| Intermediate | |  |  |  |  |  |  |  |  |  |  |  |  |
|  | Q1 | Reference |  | Reference | | Reference | | Reference | | Reference | | Reference | |
|  | Q2 | 1.05(1.00,1.10) | | 1.04 (0.99, 1.10) | | 1.05 (1.00, 1.10) | | 1.05 (1.00, 1.10) | | **1.07 (1.02, 1.12)** | | **1.07 (1.03, 1.13)** | |
|  | Q3 | **1.20(1.15,1.26)** | | **1.19 (1.13, 1.26)** | | **1.20 (1.15, 1.26)** | | **1.18 (1.12, 1.24)** | | **1.20 (1.14, 1.26)** | | **1.21 (1.15, 1.27)** | |
| High |  |  |  |  |  |  |  |  |  |  |  |  |  |
|  | Q1 | Reference |  | Reference | | Reference | | Reference | | Reference | | Reference | |
|  | Q2 | **1.12(1.04,1.20)** | | **1.12 (1.04, 1.22)** | | **1.12 (1.04, 1.20)** | | **1.11 (1.02, 1.20)** | | **1.15 (1.07, 1.24)** | | **1.15 (1.07, 1.24)** | |
|  | Q3 | **1.23(1.14,1.33)** | | **1.26 (1.16, 1.37)** | | **1.23 (1.14, 1.33)** | | **1.22 (1.12, 1.32)** | | **1.23 (1.14, 1.32)** | | **1.25 (1.15, 1.34)** | |

CHD, coronary heart disease; CI, confidence interval; HR, hazard ratio.

Sensitivity analysis 1, Exclude baseline diabetic patients; Sensitivity analysis 2, Additionally adjusted for environmental stressors, including residential noise pollution, residential particulate air pollution (pm10), and well-being in life; Sensitivity analysis 3, Additionally excluded participants with incident CVD in the previous 2 years; Sensitivity analysis 4, Additionally adjusted for diuretic use; Sensitivity analysis 5, Using standardized weighted GRS. Significant results are indicated in bold (*P* < 0.05).

## Table S24. Additive interaction between urate and GRSs on CVDs risk.

| Outcome | Urate | Genetic risk | RERI | AP |
| --- | --- | --- | --- | --- |
| CHD | Q2 | Intermediate | 0.07 (-0.05, 0.19) | 0.06 (-0.04, 0.16) |
| CHD | Q2 | High | **0.22 (0.06, 0.38)** | **0.14 (0.04, 0.23)** |
| CHD | Q3 | Intermediate | **0.13 (0.02, 0.25)** | **0.10 (0.01, 0.18)** |
| CHD | Q3 | High | **0.28 (0.12, 0.43)** | **0.15 (0.07, 0.24)** |
| IS | Q2 | Intermediate | -0.19 (-0.51, 0.13) | -0.15 (-0.39, 0.09) |
| IS | Q2 | High | -0.12 (-0.51, 0.27) | -0.08 (-0.36, 0.19) |
| IS | Q3 | Intermediate | -0.05 (-0.34, 0.25) | -0.03 (-0.23, 0.17) |
| IS | Q3 | High | 0.06 (-0.31, 0.43) | 0.04 (-0.19, 0.26) |

AP, attributable proportion due to interaction; CHD, coronary heart disease; CI, confidence interval; CVD, cardiovascular disease; HR, hazard ratio; IS, ischemic stroke; RERI, relative excess risk due to interaction.

Multivariable model was adjusted for age, sex, income score, body mass index(BMI), qualification, smoking status, alcohol status, total physical activity level, duration of sleep, fruit consumption, processed meats consumption, vegetables consumption, fishes consumption, tea consumption, coffee consumption, family history of heart diseases or stroke (only in the corresponding analysis), prevalent hypertension, prevalent diabetes, high-density lipoprotein cholesterol (HDLc), low-density lipoprotein cholesterol (LDLc), use of antihypertensive drugs, use of antihyperlipidemic drugs and use of antidiabetic drugs. The estimates of RERI and AP were calculated based on the reference group with the lowest tertile of urate and low genetic risk. Significant results are indicated in bold.

## Table S25. Joint associations of urate and outcome specific GRS with CVDs using standardized GRS method.

| Group | CHD | | IS | |
| --- | --- | --- | --- | --- |
|  | HR (95% CI) | *P* | HR (95% CI) | *P* |
| Low genetic risk |  |  |  |  |
| Q1 | 1.00 (reference) | reference | 1.00 (reference) | reference |
| Q2 | 1.01 (0.93, 1.10) | 0.736 | 1.21 (1.01, 1.46) | 0.040 |
| Q3 | 1.11 (1.02, 1.20) | 0.013 | 1.26 (1.05, 1.51) | 0.012 |
| Intermediate genetic risk | |  |  |  |
| Q1 | 1.14 (1.06, 1.23) | <0.001 | 1.25 (1.07, 1.48) | 0.007 |
| Q2 | 1.23 (1.14, 1.32) | <0.001 | 1.28 (1.09, 1.50) | 0.003 |
| Q3 | 1.38 (1.29, 1.49) | <0.001 | 1.47 (1.25, 1.73) | <0.001 |
| High genetic risk |  |  |  |  |
| Q1 | 1.41 (1.29, 1.53) | <0.001 | 1.34 (1.10, 1.62) | 0.003 |
| Q2 | 1.64 (1.52, 1.78) | <0.001 | 1.43 (1.20, 1.72) | <0.001 |
| Q3 | 1.79 (1.66, 1.93) | <0.001 | 1.66 (1.40, 1.98) | <0.001 |

CHD, coronary heart disease; CI, confidence interval; CVD, cardiovascular disease; HR, hazard ratio. Q1, the first tertile of serum urate; Q2, the second tertile of serum urate; Q3, the third tertile of serum urate.

Multivariable model was adjusted for age, sex, income score, Body mass index(BMI), qualification, smoking status, alcohol status, total physical activity level, duration of sleep, fruit consumption, processed meats consumption, vegetables consumption, fishes consumption, tea consumption, coffee consumption, family history of heart diseases or stroke (only in the corresponding analysis), prevalent hypertension, prevalent diabetes, high-density lipoprotein cholesterol (HDLc), low-density lipoprotein cholesterol (LDLc), use of antihypertensive drugs, use of antihyperlipidemic drugs and use of antidiabetic drugs.

# Supplemental Figures


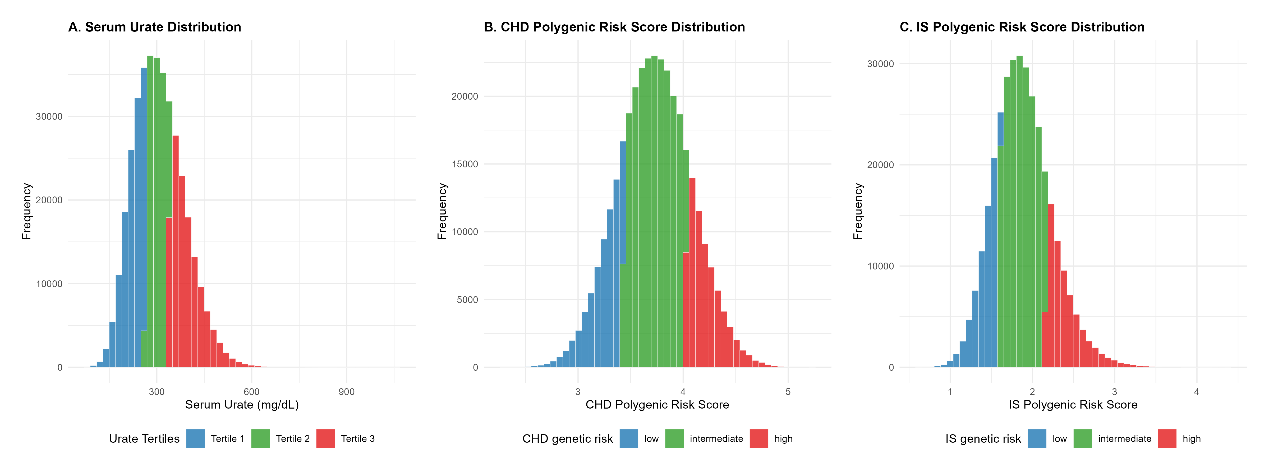


## Figure S1. Distribution of serum urate and polygenic risk scores.

## Figure S2. CKD risk defined by eGFR and ACR.

Green: low risk; Yellow: moderately increased risk; Orange: high risk; Red: very high risk. eGFR, estimated glomerular filtration rate; ACR, albumin-creatinine ratio.

##
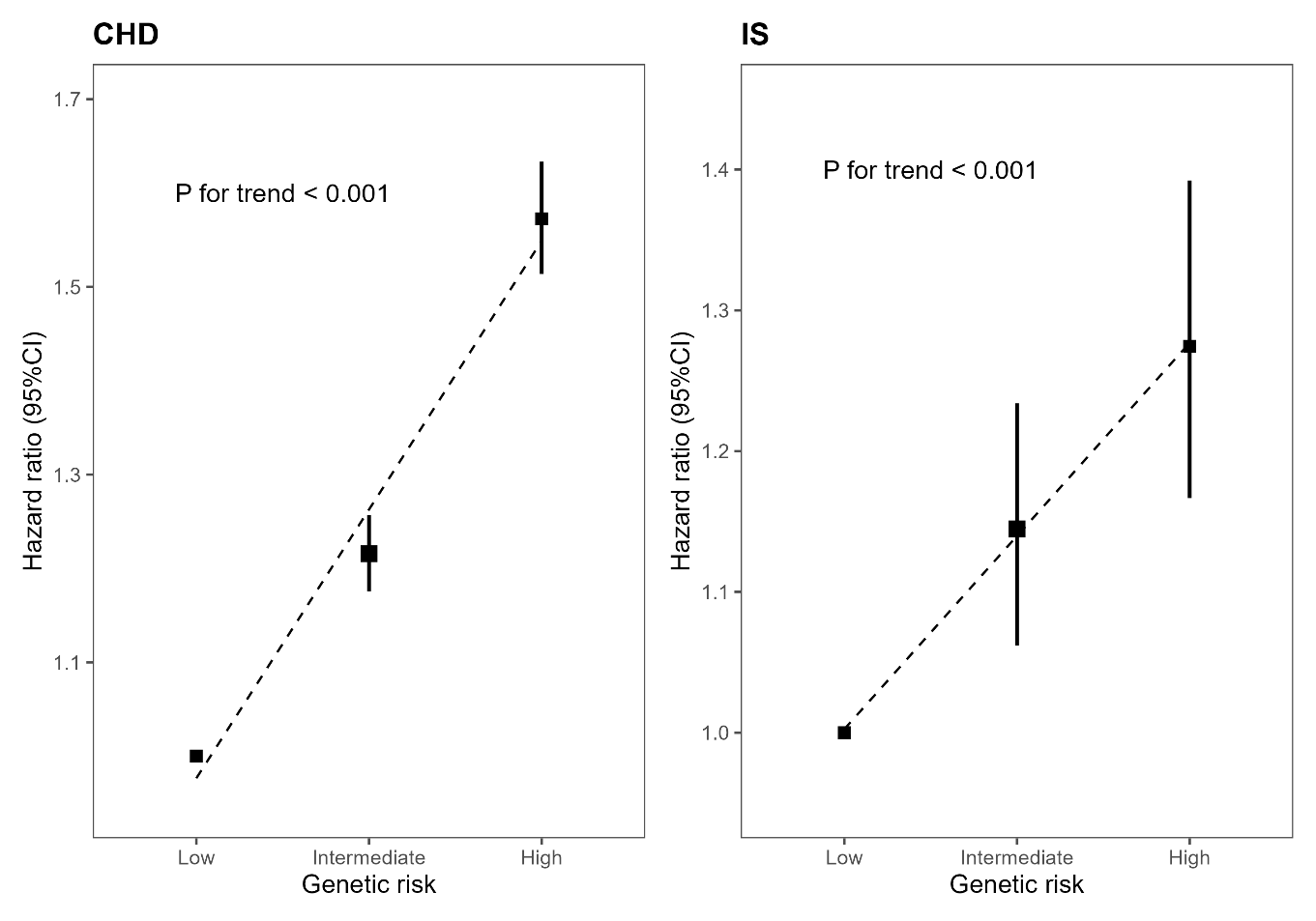
Figure S3. Incident risk of cardiovascular diseases according to genetic risk among 343,054 European ancestry participants.

Multivariable model was adjusted for age, sex, income score, body mass index(BMI), qualification, smoking status, alcohol status, total physical activity level, duration of sleep, fruit consumption, processed meats consumption, vegetables consumption, fishes consumption, tea consumption, coffee consumption, family history of heart diseases or stroke (only in the corresponding analysis), prevalent hypertension, prevalent diabetes, high-density lipoprotein cholesterol (HDLc), low-density lipoprotein cholesterol (LDLc), use of antihypertensive drugs, use of antihyperlipidemic drugs and use of antidiabetic drugs. CHD, coronary heart disease; CI, confidence interval; CVD, cardiovascular disease; HR, hazard ratio; IS, ischemic stroke.
